# Phenotypic Consequences of Copy Number Variation: Insights from Smith-Magenis and Potocki-Lupski Syndrome Mouse Models

**DOI:** 10.1371/journal.pbio.1000543

**Published:** 2010-11-23

**Authors:** Guénola Ricard, Jessica Molina, Jacqueline Chrast, Wenli Gu, Nele Gheldof, Sylvain Pradervand, Frédéric Schütz, Juan I. Young, James R. Lupski, Alexandre Reymond, Katherina Walz

**Affiliations:** 1 Center for Integrative Genomics, University of Lausanne, Lausanne, Switzerland; 2 Centro de Estudios Científicos (CECS), Valdivia, Chile; 3 Molecular & Human Genetics, Baylor College of Medicine, Houston, Texas, United States of America; 4 Swiss Institute of Bioinformatics (SIB), Lausanne, Switzerland; 5 John P. Hussman Institute for Human Genomics, University of Miami Miller School of Medicine, Miami, Florida, United States of America; 6 CIN (Centro de Ingeniería de la Innovación del CECS), Valdivia, Chile; 7 Pediatrics, Baylor College of Medicine, Houston, Texas, United States of America; 8 Texas Children's Hospital, Houston, Texas, United States of America; Medical Research Council Human Genetics Unit, United Kingdom

## Abstract

A large fraction of genome variation between individuals is comprised of submicroscopic copy number variation of genomic DNA segments. We assessed the relative contribution of structural changes and gene dosage alterations on phenotypic outcomes with mouse models of Smith-Magenis and Potocki-Lupski syndromes. We phenotyped mice with 1n (Deletion/+), 2n (+/+), 3n (Duplication/+), and balanced 2n compound heterozygous (Deletion/Duplication) copies of the same region. Parallel to the observations made in humans, such variation in gene copy number was sufficient to generate phenotypic consequences: in a number of cases diametrically opposing phenotypes were associated with gain versus loss of gene content. Surprisingly, some neurobehavioral traits were not rescued by restoration of the normal gene copy number. Transcriptome profiling showed that a highly significant propensity of transcriptional changes map to the engineered interval in the five assessed tissues. A statistically significant overrepresentation of the genes mapping to the entire length of the engineered chromosome was also found in the top-ranked differentially expressed genes in the mice containing rearranged chromosomes, regardless of the nature of the rearrangement, an observation robust across different cell lineages of the central nervous system. Our data indicate that a structural change at a given position of the human genome may affect not only locus and adjacent gene expression but also “genome regulation.” Furthermore, structural change can cause the same perturbation in particular pathways regardless of gene dosage. Thus, the presence of a genomic structural change, as well as gene dosage imbalance, contributes to the ultimate phenotype.

## Abstract 2

The characterization of mice with different number of copies of the same genomic segment shows that structural changes influence the phenotypic outcome independently of gene dosage.

## Author Summary

Mammalian genomes contain many forms of genetic variation. For example, some genome segments were shown to vary in their number of copies between individuals of the same species, i.e. there is a range of number of copies in the normal population instead of the usual two copies (one per chromosome). These genetic differences play an important role in determining the phenotype (the observable characteristics) of each individual. We do not know, however, if such influences are brought about solely through changes in the number of copies of the genomic segments (and of the genes that map within) or if the structural modification of the genome *per se* also plays a role in the outcome. We use mouse models with different number of copies of the same genomic region to show that rearrangements of the genetic materials can affect the phenotype independently of the dosage of the rearranged region.

## Introduction

Copy number variation (CNV) of genomic segments among phenotypically normal human individuals was recently shown to be surprisingly frequent [1],[2]. It covers a large proportion of the human genome and encompasses thousands of genes [3],[4]. About 58,000 human CNVs from approximately 14,500 regions (CNVRs) have been identified to date (http://projects.tcag.ca/variation/). They contribute to genetic variation and genome evolution [5]–[8] by modifying the expression of genes mapping within the CNV and in its flanks [9]–[13]. Consistently, initial cases of adaptive CNV alleles under positive selection were recently uncovered [14] and several structural variants were shown to be associated with “genomic disorders” [15]–[17] and susceptibility to disease (reviewed in [7],[18]–[21]). For example, a microdeletion and its reciprocal microduplication at chromosomal band 17p11.2 were shown to be associated with Smith-Magenis (SMS; OMIM#182290) and Potocki-Lupski syndromes (PTLS; OMIM#610883), respectively [22]–[24]. The Retinoic Acid Induced gene 1 (*RAI1*; GeneID: 10743) is thought to be the main dosage-sensitive gene within this genomic interval. Consistently, SMS patients with only *RAI1* mutation have been identified [25]–[28]. However, accumulating evidence indicates that other factors also contribute to the spectrum of clinical findings in patients. For example, SMS patients with *RAI1* mutations are less likely than SMS patients with the deletion to be short and suffer from hearing loss, cardiovascular, and renal tract abnormalities. On the other hand, they are at higher risk for obesity [29]–[33]. Mouse models of these syndromes were generated. These engineered animals recapitulate several of the multiple phenotypes present in the human patients. The SMS mice show craniofacial abnormalities, obesity, overt seizures, hypoactivity levels, and circadian rhythm anomalies, while the PTLS model is underweight and presents hyperactivity, learning and memory deficiencies, and social impairment [11],[27],[34],[35].

We took advantage of these models and of a third strain that is a compound heterozygote balanced for copy number—it harbors the SMS deletion on one allele and the PTLS duplication on the other—to tease apart the phenotypic consequences of gene dosage alterations versus genomic structural changes.

## Results

**Figure 1:**
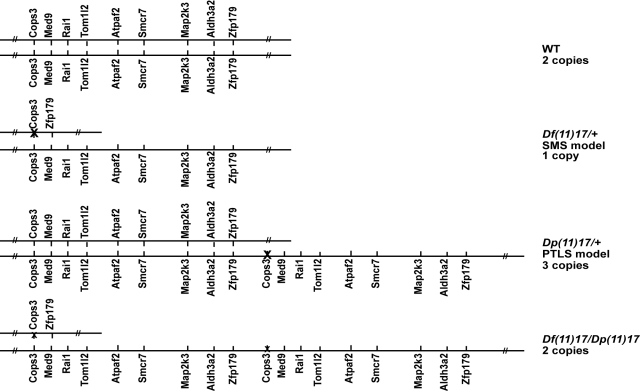
SMS and PTLS mouse models. Schematic representation of the mouse chromosome 11 B2 region syntenic to the SMS and PTLS critical region to compare the genotypes of the four strains used in this report (adapted from [34]). Only a few genes of the engineered region are displayed. The region contains the following loci, whose expression is profiled by 70 different probesets: *Cops3*, *Nt5m*, *Med9*, *Rasd1*, *Pemt*, *Rai1*, *Srebf1*, *Tom1l2*, *Lrrc48*, *Atpaf2*, *4933439F18Rik*, *Drg2*, *Myo15*, *Alkbh5*, *AW215868*, *Llgl1*, *Flii*, *Smcr7*, *Top3a*, *Smcr8*, *Shmt1*, *Dhrs7b*, *Tmem11*, *Gtlf3b*, *Gtlf3a*, *Map2k3*, *Kcnj12*, *Tnfrsf13b*, *Usp22*, *Aldh3a1*, *Aldh3a2*, *Slc47a2*, *Slc47a1*, and *Zfp179* (a.k.a. *Rnf112*) (for GeneIDs, see Materials and Methods). The *Cops3* and *Zfp179* loci were used as anchoring points to engineer the rearrangement [34], thus their number of copies does not correlate with the number of copies of the region. Furthermore, some copies of *Cops3* (indicated by an X) were inactivated in the process [34].

The functional impact of CNV of a given genomic interval remains unstudied at a genome-wide scale. Such a global assessment is achievable nowadays using the mouse as a model organism. Mouse models of the Smith-Magenis and Potocki-Lupski syndromes carry a deletion (strain *Df(11)17/+*) and its reciprocal duplication (*Dp(11)17/+*), engineered rearrangements involving the syntenic genomic regions at band MMU11B2, respectively [11],[22]–[24],[27],[28],[30],[34],[35]. These heterozygous mice and their wild type littermates (*+/+*) allow the study of the influence of one, two, and three copies of the same CNV in an otherwise identical genomic background (see below). A fourth strain (*Df(11)17/Dp(11)17*) obtained by mating the *Dp(11)17/+* and *Df(11)17/+* animals enables the generation of genomically balanced mice with two copies of that same CNV in *cis*, while they are in *trans* in *+/+* animals (see Figure 1 for a schematic representation of the four genotypes).

### Influence of Gene Dosage and Structural Changes on the Phenotypic Outcome

To investigate the phenotypic outcome of modifying gene dosage or of maintaining gene dosage but with a structural change, we assessed 14 different phenotypes in the four different mouse genotypes (i.e., 1n, 2n, 3n, and 2n compound heterozygote) (Table 1). The decreased embryonic survival, craniofacial abnormalities, overt seizures, and altered neuromotor function observed in *Df(11)17/+* and the learning and memory impairments shown by *Dp(11)17/+* animals were absent in the genetically balanced *Df(11)17/Dp(11)17* mice (summarized in Table 1; for details see Text S1, Figures S1–S2 and Table S1). Likewise, the significant differences in body weight and abdominal fat found in the SMS and the PTLS mouse models when compared to *+/+* animals were absent in *Df(11)17/Dp(11)17* (Text S1 and Figure S3). Furthermore, we found that “backing out of the test tube,” when confronted by a wild type mouse, was only correlated with copy numbers but not with structural changes *per se* (Text S1 and Figure S4). A summary of phenotypic differences between *Rai1 +/*− and *Df(11)17/+* mice can be found in Text S1.

**Table 1:** Not all phenotypes are recovered with the correct gene dosage in the region.

Genotype [Gene copy number within this genomic interval]:	*Dp(11)17/+* [3n]	*Df(11)17/+* [1n]	Wild type [2n]	*Df(11)17/Dp(11)17* [2n]
*Phenotype:*				
Viability	normal	reduced	normal	normal
Craniofacial abnormalities	absent	present (99%)	absent	absent
Overt seizures	absent	present (9%)	absent	absent
Body weight	*underweight*	*overweight*	normal	normal
Abdominal fat	*underweight (1%)*	*overweight (3%)*	normal (2%)	normal (2%)
**Anxiety (plus maze)**	***elevated***	***decreased***	**normal**	**decreased**
Sociability	normal	normal	normal	normal
**Preference for social novelty**	***decreased***	***increased***	**normal**	**decreased**
Dominant behavior	*increased*	*decreased*	normal	normal
Dowel test (number of falls)	normal	increased	normal	normal
Hanging ability	normal	decreased	normal	normal
Rotating rod	abnormal performance	abnormal performance	normal	normal
**Activity levels (open field)**	***elevated***	***decreased***	**normal**	**elevated**
Learning and memory (conditioned fear)	impaired	normal	normal	normal

The observed and systematically examined phenotypes for the four experimentally tested genotypes are summarized with the results found for each genotype. In bold type are those phenotypes that were not rescued in *Df(11)17/Dp(11)17* mice. Italics represent opposing phenotypes in *Df(11)17/+* versus *Dp(11)17/+* mice. Some phenotypes were previously reported in [11],[27],[34],[35].

**Figure 2:**
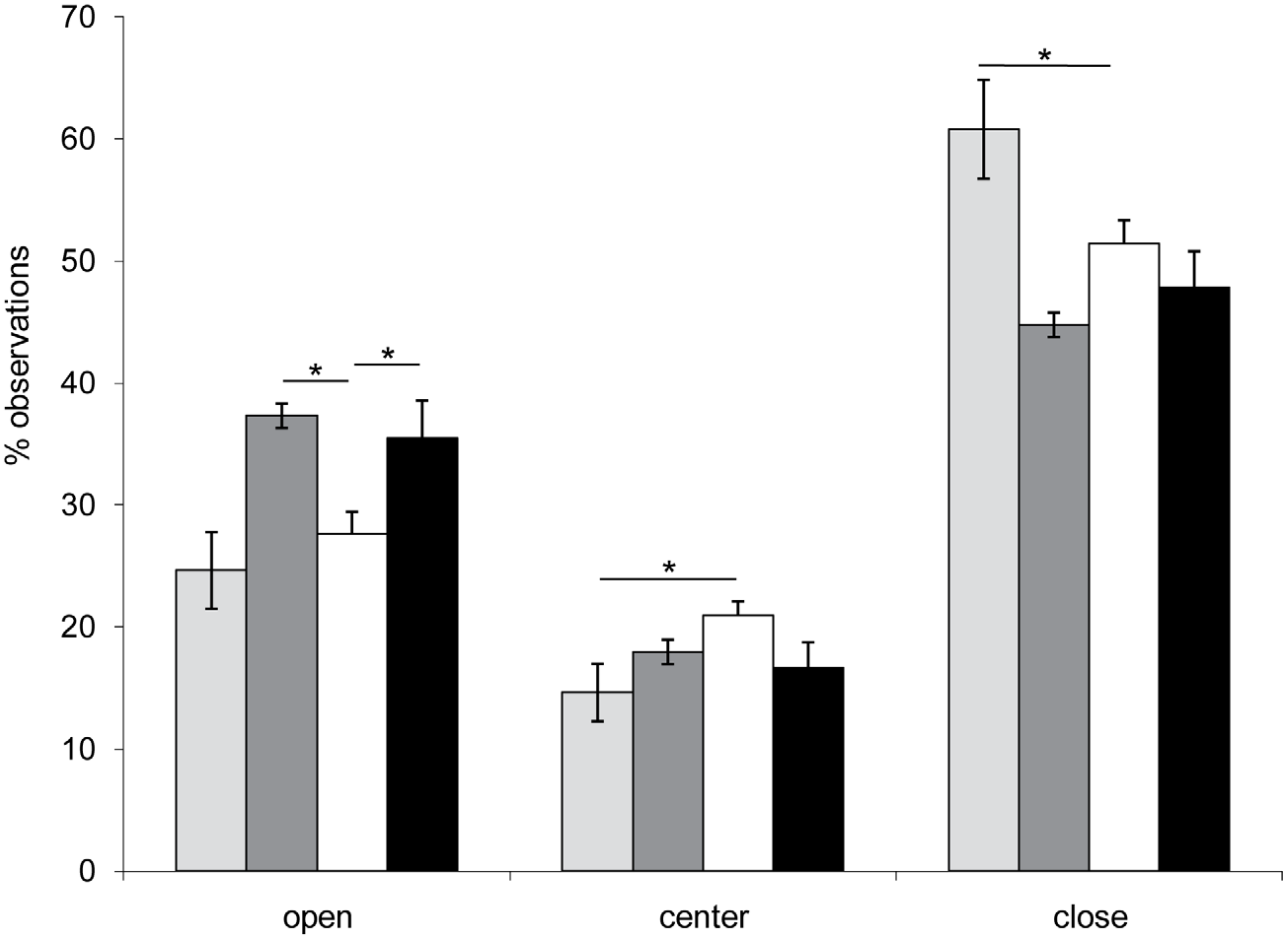
Anxiety in the plus maze test is not normalized with the correction of the gene copy number. The percentage of observations in each arm or the center of the plus maze is represented. Light grey columns: *Dp(11)17/+* (*N* = 19); dark grey columns: *Df(11)17/+* (*N* = 20); white columns: +/+ (*N* = 27); and black columns: *Df(11)17/Dp(11)17* (*N* = 22). Values represent mean ± SEM. The asterisk denotes significant differences (* *p*<0.05).

Anxiety was found increased in *Dp(11)17/+* mice in the elevated plus maze test [11]. We found an overall significant difference in the percentage of observations in the open arms (*F*_(3, 87)_ =  5.9; *p* = 0.001) and closed arms (*F*_(3, 87_ = 8; *p*<0.0001). Post-hoc analysis showed that *Dp(11)17/+* mice spend significantly more time in the closed arms (62.1%±3%) than their wild type littermates (51%±1.9%) (*p* = 0.002). In contrast, the percentage of observations in the open arms was significantly increased for *Df(11)17/+* mice (37%±2.5%), when compared with *+/+* animals (29%±1.9%) (*p* = 0.023). The percentage of observations in the open arm was also significantly increased for *Df(11)17/Dp(11)17* mice (36%±2.2%), when compared with *+/+* (*p* = 0.045), however the *p* value is in the borderline range. The number of observations of *Df(11)17/Dp(11)17* mice in the center and the close arm was always smaller than that of wild type. This is concordant with what we observed for *Df(11)17/+* mice. While none of these differences are significant, both *Df(11)17/+* and *Df(11)17/Dp(11)17* mice behave similarly. No significant differences were observed when *Df(11)17/Dp(11)17* were compared to the *Df(11)17/+* mice (*p*>0.05). These results indicate that dosage of genes mapping within the engineered genomic interval is associated with the levels of anxiety in mice, since the gain or loss of genetic material are giving opposite phenotypes. However, structural changes play a role, as restoration of the number of copies (2n in *cis*) does not rescue the phenotype (Figure 2 and Table 1). This observation was similar to what was found for activity levels in the open field (Table1) [27].

**Figure 3:**
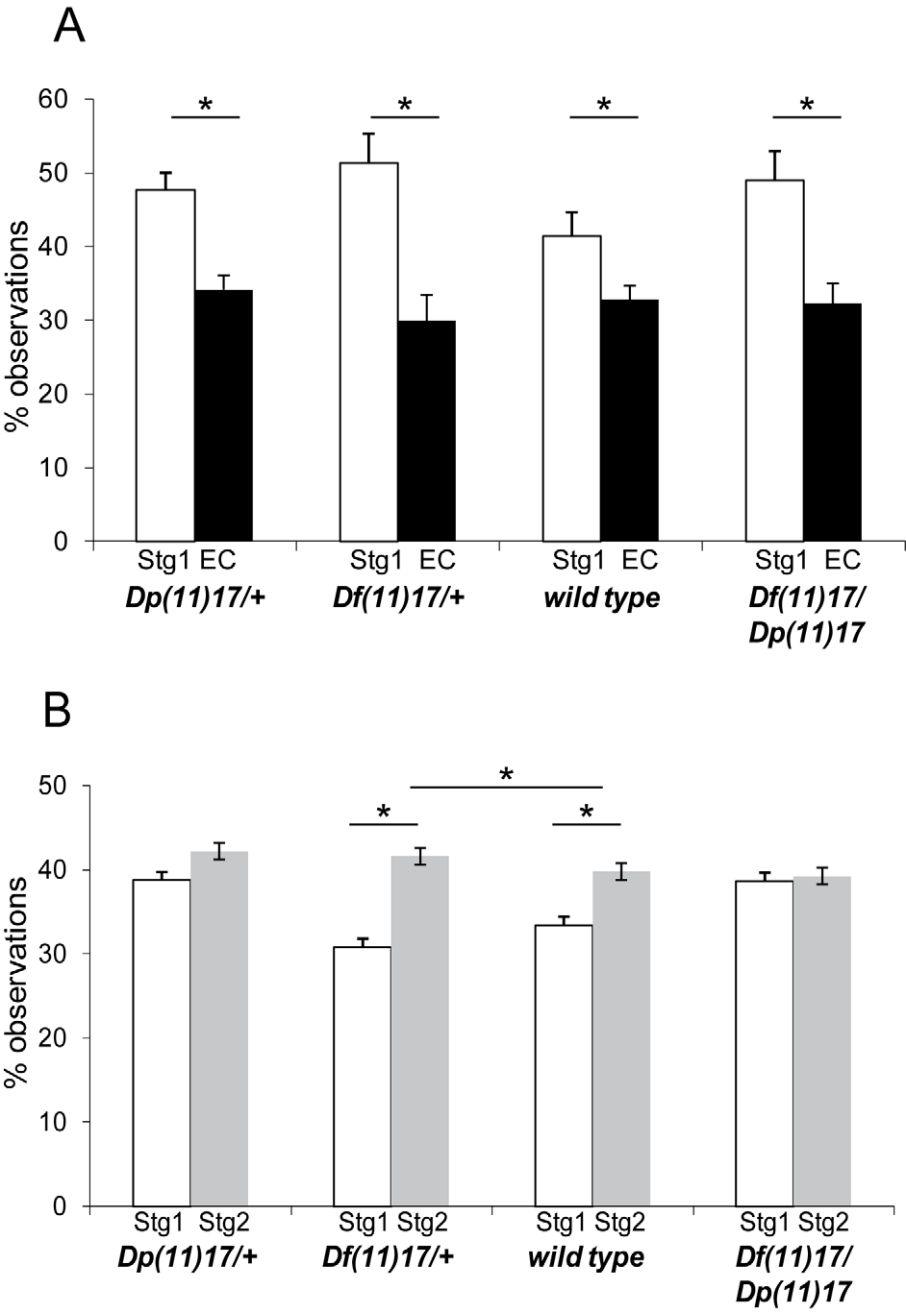
Some social behaviors are dependent on the presence of genomic rearrangements. (A) Percentage of observations in the chamber side with stranger 1 (Stg1, white columns) or with the empty container (EC, black columns) during the sociability test is shown for the four different groups of mice. (B) Percentage of observations in the chamber side with stranger 1 (Stg1, white columns) or with stranger 2 (Stg2, grey columns) during the preference for social novelty test is depicted. For each genotype the number of mice tested was: *N* = 21 for *Dp(11)17/+*, *N* = 23 for *Df(11)17/+*, *N* = 28 for +/+, and *N* = 22 for *Df(11)17/Dp(11)17* mice. The mean ± S.E.M. values are presented. Asterisk denotes significantly differences (* *p*<0.05).

*Dp(11)17/+* mice showed a subtle impairment in the preference of a social target versus an inanimate target and a clear impaired preference for social novelty when compared to +/+ mice [11] in the three-chamber test [36] that is based on the tendency of a subject mouse to approach and engage in social interaction with an unfamiliar mouse. We performed this test in the four different groups of purebred mice with distinct CNV genotypes. The analysis of the sociability part of the test showed a significant effect of chamber side (*F*_(1, 90)_ = 38.99, *p*<0.0001). Post-hoc analysis demonstrated that mice from all analyzed genotypes spend more time in the chamber side that contains the stranger 1 versus the side with the empty container (*p*<0.01 in all cases) (Figure 3A). In the preference for social novelty data, we observed a significant difference for chamber side (*F*_(1, 90)_ = 9.6, *p* = 0.0025) and genotype (*F*_(3, 90)_ = 5.74, *p* = 0.0012). Post-hoc analysis revealed that wild type (*p* = 0.04) and *Df (11)17/+* mice (*p* = 0.0002) tend to spend significantly more time with stranger 2 than with stranger 1, but *Dp(11)17/+* and *Df(11)17/Dp(11)17* mice spent the same amount of time with stranger 1 and stranger 2 (*p* = 0.37 and 0.87, respectively). Moreover, when +/+ mice were compared with the other three genotypes we found that they spend significantly less time in the side of the stranger 1 than the *Dp(11)17/+* mice (*p* = 0.0002) and *Df(11)17/Dp(11)17* mice (*p* = 0.0003), but no significant differences were found when compared to *Df(11)17/+* mice (*p*>0.05). In aggregate, these results suggest that gene copy number variation is playing a role in the preference to social novelty and that the duplication or deletion of this genomic interval is giving an opposite phenotype. Surprisingly, the response to social novelty is also modified in *Df(11)17/Dp(11)17* mice, notwithstanding that gene dosage is normalized (Figure 3B and Table 1), suggesting that genomic structural changes are playing a role in this phenotypic outcome.

### The Expression of Genes Mapping within the Engineered Interval Is Modified

The phenotypic findings in mice prompted us to assess the effect of changing the number of copies of the SMS/PTLS CNV on tissue transcriptomes. We analyzed genome-wide expression levels in five organs affected in human patients (cerebellum, heart, kidney, testis, and hippocampus) from adult male individuals (at least three animals of each of the strains carrying one, two in *trans*, two in *cis*, and three copies of the MMU11B2 region; see Materials and Methods).

**Figure 4:**
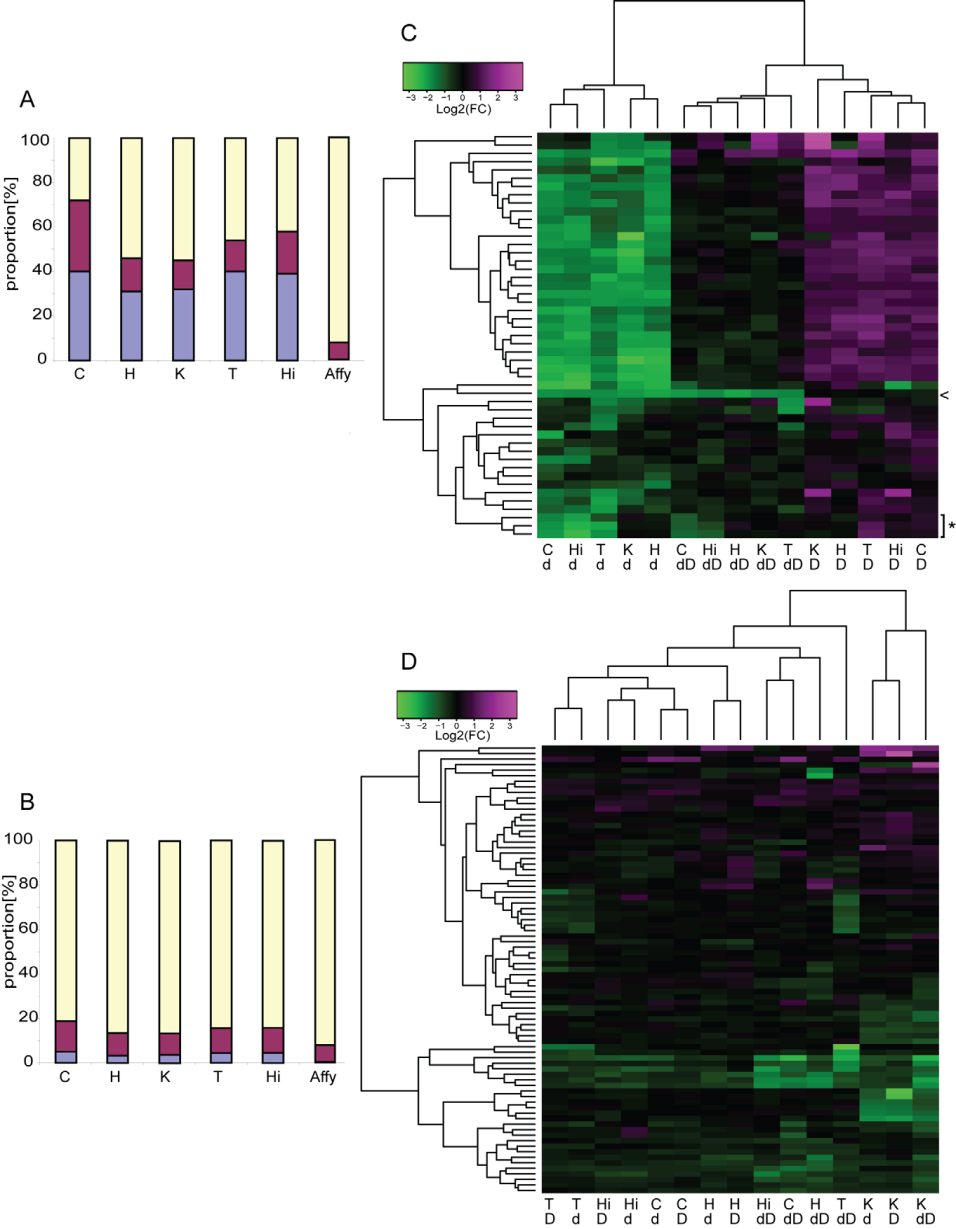
Differentially expressed genes in SMS and PTLS mouse models. Distribution of the mapping regions of the top 100 (A) and top 1,000 ranked (B) most differentially expressed transcripts in the cerebellum (C), heart (H), kidney (K), testis (T), and hippocampus (Hi) or present on the array (Affy) of *Df(11)17/+* (SMS model, 1n), *Dp(11)17/+* (PTLS model, 3n), *Df(11)17/Dp(11)17* (2n compound heterozygote), and *+/+* (2n) mice (Most-diff dataset, see Figure 1 for a schematic representation of the mouse 11 B2 region of the different mouse models). Proportion of transcripts mapping to the SMS/PTLS rearranged interval (purple), the remainder of mouse chromosome 11 (burgundy), and elsewhere (yellow). Transcripts mapping to the rearranged interval and to the remainder of mouse chromosome 11 are both statistically overrepresented in all tested tissues (all *p*<1×10^−4^). Heatmap of the changes in expression levels of the 49 Most-diff transcripts mapping to the SMS/PTLS rearranged interval (C) and the remainder of mouse chromosome 11 (81 transcripts) (D) measured in *Df(11)17/+* (d), *Dp(11)17/+* (D), and *Df(11)17/Dp(11)17* (dD) mice as compared to +/+ individuals in cerebellum (C), heart (H), kidney (K), testis (T), and hippocampus (Hi). The arrowhead and asterisk denote *Cops3* and *Zfp179* transcripts, respectively. These transcripts were used as anchors in the strain engineering process, thus they are not present in the same number of copies than other SMS/PTLS genes in the mice models (see Figure 1 and text for details).

We ranked and chromosomally mapped the most differentially expressed transcripts. As anticipated, we observed in each of the analyzed tissues a significant overrepresentation of transcripts mapping to the rearranged interval (which we named SMS/PTLS genes; see legend of Figure 1 or Materials and Methods for a complete list of loci mapping to the engineered interval) amongst the top 100 (31 to 40 transcripts depending on the tissue) and top 1,000 (33 to 50 transcripts) most differentially expressed transcripts (all *p*<1×10^−4^, tested with permutations; Figure 4A–B). The expression levels of the transcripts, which vary in number of copies amongst the different strains, are compared in Figure 4C. We found a positive correlation between gene dosage and expression consistent with partial results already published [11]. These transcripts are expressed on average at 66%±15% of the level measured in wild type in *Df(11)17/+* (one copy) and 138%±29% in *Dp(11)17/+* animals (three copies). In particular, the expression levels of the murine orthologs of the two genes *RAI1* (GeneID: 10743) and *SREBF1* (6720), which were associated with schizophrenia [37]–[39], a phenotype absent from SMS and PTLS patients [33],[40],[41], show a strong relationship with gene dosage. The SMS/PTLS genes are, however, unchanged in *Df(11)17/Dp(11)17* mice (1.02-fold (SD = 0.16) more, two copies in *cis*) compared to normal controls (two copies in *trans*), analogous to results recently obtained from cell lines of a man who carried a 22q11 deletion on one allele and a reciprocal duplication on the other allele [42]. Note that the *loxP* site inclusions necessary for the mouse engineering induced the loss-of-function of one *Cops3* copy (GeneID: 26572) (Figure 1) [34], thus *Df(11)17/Dp(11)17* and *Dp(11)17/+* animals have only a single and two active copies of this gene, respectively. Consistently, we found *Cops3* relative expression level to be downregulated in the compound heterozygous animals and unchanged in the PTLS mouse model (Figure 4C). The *Df(11)17/+* and *Df(11)17/Dp(11)17* strains carry two and three copies of *Zfp179* (a.k.a. *Rnf112*, GeneID: 22671), respectively (Figure 1 and 4C), thus this gene could be considered in the “flanking” genes category in some strains (see below).

To confirm the transcriptome profiling results, we independently measured by Taqman quantitative PCR the relative expression levels of 43 genes in the hippocampus and cerebellum of males (*N* = 3) and females (*N* = 3) and the cortex, liver, and lung of female mice (*N* = 3) of the *+/+*, *Dp(11)17/+*, and *Df(11)17/+* genotypes. The list of genes and assays used are presented in Table S2. They map either centromeric, within, or telomeric to the rearranged region. We found good reproducibility of the data for the three genes that were quantified with two different Taqman assays (Figure S5). Likewise, we noted a robust correlation between the Taqman and expression microarray results (correlation coefficient, R^2^ = 0.87; Figure S5A). Furthermore, the assays performed on female tissues demonstrated that the above described influences on the expression levels of genes situated within the rearrangement are not restricted to one sex and to the five tissues monitored by microarray (Figure S5B and S5C). Thus, the altered expression of SMS/PTLS genes are most probably relevant to the development of the phenotypic manifestations of PTLS and SMS mouse models that are absent in the *Df(11)17/Dp(11)17* animals.

### The Expression of Normal Copy Number Genes Mapping on MMU11 Is Modified

A second category of transcripts, those that map to the rest of mouse chromosome 11 (MMU11 genes), was significantly enriched within the top 1,000 most differentially expressed transcripts in all five tissues (all *p*<1×10^−4^, 97 to 138 transcripts, Figure 4B; “Most-diff” set of data, see below and Materials and Methods). This “flanking effect” might not be an effect of structural changes but could potentially be caused by linkage disequilibrium between the engineered interval and flanking polymorphisms. Consistently, retention of large blocks from the parental strain through genetic selection even after repeated backcrossing has been reported [43]–[45].

The SMS and PTLS mouse models were generated from a different genetic background (i.e., the AB2.2 ES cell line derived from a 129S5 mouse, see [34] for details) and were backcrossed for 12 generations to C57BL/6J-*Tyr^c-Brd^*. Genotyping of the entire length of MMU11 revealed that, whereas the region proximal to the engineered interval had recombined, the distal section had either only partially or not recombined at all to the C57BL/6J background in *Dp(11)17/+* and *Df(11)17)/+*, respectively (Figure S6A). These sequence variants may have a significant impact on microarray-based transcriptome profiling [46]–[48]. For example, almost half of the reported 100 most significant *cis*-acting expression QTLs could be attributed to sequence diversity in probe regions in [46].

We thus devised a strategy to identify and discard the transcripts that could possibly be influenced by their 129S5 genetic makeup rather than by the modification of the number of copies of the CNV. As we found that 129S5 and 129S2 mice were genetically identical at all tested loci from the SMS/PTLS engineered interval to the telomere, we thought to use expression data previously established in our laboratory with the same microarray platform (GEO Series accession number: GSE10744) [12] to identify the transcripts that show a different level of expression between 129S2 and C57BL/6J animals in at least one of six major tissues (brain, liver, testis, kidney, lung, and heart) (false discovery rate<0.1; corrected for multiple testing) and that thus should be removed from our analysis (see Materials and Methods, Figure S7). This allowed establishment of a restricted set of data, named Most-diff-restricted, in which these transcripts were excluded (the unrestricted set was named “Most-diff”; see Materials and Methods). Within this constrained set, we found again that the SMS/PTLS transcripts were significantly enriched within the top 1,000 most differentially expressed transcripts in all five tissues analyzed (Most-diff-restricted set: all *p*<1×10^−4^, 26 to 40 transcripts). Similarly, the transcripts that map to the rest of the MMU11 chromosome were significantly enriched within the top 1,000 most differentially expressed transcripts in the cerebellum and hippocampus (Most-diff-restricted set: *p*<1×10^−3^, 94 and 103 transcripts, respectively) but not in the other three monitored tissues.

One could argue that this class of transcripts is still overrepresented in the two neuronal tissues because we were unsuccessful in identifying and discarding all transcripts that are influenced by linkage disequilibrium. Hence, to further assess a potential bias caused by the linkage disequilibrium between the engineered interval and flanking polymorphisms, we compared in three different tissues (cerebellum, kidney and testis) the relative expression of genes before and after their recombination to a C57BL6/J homozygous genetic background. We measured by quantitative PCR the relative expression levels of genes showing significant differences in expression between *Dp(11)17/+* and *+/+* in the microarray profiling experiments (see above) and mapping to the 11:76843886–92963733 interval in *Dp(11)17/+* mice after 12 and 17 backcrosses (129S5/C57BL6/J heterozygous and C57BL6/J/C57BL6/J homozygous background, respectively) and compared it to that of wild type littermates (Figure S6A). The different assays are presented in Table S3. We found that 7 out of 14 (50%), 8 out of 16 (50%) and 12 out of 16 (75%) of the genes we studied in testis, kidney, and cerebellum, respectively, showed a change in expression level between the PTLS model and controls even after recombination, suggesting that the observed differences in expression of these genes are independent of the genetic background and not caused by linkage disequilibrium of the engineered region (Figure S6B–S6D). These results justify the strategy used above to discard 129 out of 248 probesets that could possibly be influenced by their 129S5 background.

Contrary to what we observed for the genes that map to the rearranged intervals, the “flanking” transcripts presented no correlation between gene dosage of the SMS/PTLS CNV and their expression levels (Figure 4C–D and Figure S8). In fact a majority (>55% within Most-diff-restricted and >80% within Most-diff) of the MMU11 transcripts showed a similar change in expression level in the *Df(11)17/+*, *Dp(11)17/+*, and *Df(11)17/Dp(11)17* animals compared to normal controls in all analyzed tissues. As an important proportion of the MMU11 genes that do not vary in number of copies appeared to be affected in a consistent manner in the engineered animals, it is unlikely that their expression is solely directly or indirectly controlled by one or a combination of the 34 genes mapping to the rearranged interval (see Figure 1 or the Materials and Methods section for the complete list of these genes). If this would have been the case we might anticipate observing opposite changes in expression in the SMS and in the PTLS mice (see above and below). Consistently, we observe similar expression levels not only in the mice with one or three copies but also in the balanced heterozygote animals with two copies in *cis* of the SMS/PTLS CNV. In this latter strain, these changes in expression levels of the MMU11 transcripts are identified, although we register no modifications of the expression levels of the SMS/PTLS transcripts (Figure 4C and Figure S8B). Similarly, the analogous changes in expression reported in the different engineered genotypes could not be explained by the retention of promoters driving the introduced selection markers, as a previously shown possible explanation we needed to control for (e.g., [49]–[51], reviewed in [52]), because different cassettes are maintained in the three different models, i.e. puromycin and neomycin resistance genes in *Df(11)17/+* and *Df(11)17/Dp(11)17* and *Hprt*, tyrosinase and *K14Agouti* genes in *Dp(11)17/+* and *Df(11)17/Dp(11)17* [34].

One mechanism explaining the observed deregulation of MMU11 transcripts might be the dissociation of these transcripts from their long-range regulatory elements, a phenomenon known as position effect [53]. If the changes in gene expression were caused by the physical separation of *cis*-acting regulatory elements mapping to the rearranged interval and MMU11 genes, we should expect an enrichment of affected genes close to the breakpoints (i.e., the *loxP* sites necessary for the mouse engineering [34]). This is only partially the case (Figure S9). In fact, we find genes with modified expression mapping on the entirety of mouse chromosome 11, for example, tens of megabases from the breakpoints, suggesting that other mechanisms of regulation might also be at play (Figure S9). We find, however, no correlation between the distance from the breakpoints and the extent of expression change (Figure S10). Many of the transcripts that show changes in relative expression appear to cluster in discrete groups along the chromosome. We tested this assumption using a modified version of the method described by Tang and Lewontin to infer significance (see Materials and Methods) [54],[55] but found no significant clustering of the modified transcripts. We thus infer that the observed clustering is simply due to the non-homogenous distribution of genes along mouse chromosome 11 (Figure S9B). Similarly, we found no significant enrichment of genes that neighbor CpG islands within the set of MMU11 CNV-affected transcripts (Most-diff-restricted: *p*<0.25; Most-diff: *p*<0.15 tested with permutations; see Materials and Methods), which could have suggested that these genes are expressed in many tissues [56]. We found, however, that the MMU11 transcripts modified in expression were expressed in a significantly greater fraction of the tissues we assessed (average 2.6, median 3) relative to other transcripts (1.8, 2; two-tailed *p*<2.2×10^−16^, Mann-Whitney *U* test). They are, however, not expressed at higher levels than their unchanged counterparts (Figure S11).

Interestingly, the two tissues that show a significant number of differentially expressed genes mapping to MMU11, i.e. hippocampus and cerebellum, are part of the central nervous system (CNS). This observation suggests that copy number changes may have more of an effect on normal copy neighboring genes expressed in the brain. Other reports have shown that genes expressed in the brain have changed less than have genes expressed in other tissues during evolution [57] and that CNV genes expressed in the brain are more tightly regulated than other CNV genes [12]. The stricter expression regulation of genes with a function in the CNS is possibly brought about by their increased interdependency through multiple feedback loops, common long-range *cis*-acting regulatory units, and/or changes in the chromatin conformation. Thus, suggesting that perturbation to such “higher order” genome organization would be more identifiable and consequential in the CNS. Consistently, the phenotypes that persist upon restoration of gene dosage, modification of activity, anxiety, and sociability levels, are most probably from a neurological origin. We identified gene(s) that are modified in their relative expression levels in the *Df(11)17/Dp(11)17* mouse (see above). The comparison of the hippocampal and cerebellar transcriptomes of these mice with that of *+/+* littermates showed that expression levels of genes involved in detection of stimuli, visual perception, as well as neuronal differentiation were modified and, thus ultimately, might be at the origin of the change in phenotypic outcome (Text S1, Table S4–S6).

Taken together our results indicate that structural changes *per se*, i.e. without changes in gene dosage, have genomic consequences on gene expression far beyond the locus whose structure is varied and that structural variation can profoundly modify the phenotypic outcome.

## Discussion

Copy Number Variants (CNVs), because of their prevalence, e.g. 10% of the mouse autosomal genome and 60% of its duplicated regions [12],[58], constitute important contributors to intraspecific genetic variation. Multiple human CNVs have been associated with diseases, susceptibility to diseases, and adaptation (reviewed in [7],[8],[18]–[20]).

We show that mouse models of Smith-Magenis and Potocki-Lupski syndromes, engineered to have one and three copies, respectively, of the mouse chromosome 11 (MMU11) band B2 region (Figure 1) present altered expression of the genes mapping within the rearranged interval and diametrically opposing phenotypes in body weight, percent fat, anxiety, preference for social novelty, dominant behavior, and activity levels (Table 1). Similarly, the deletion and reciprocal duplication of the 1q21.1 region are associated with micro- and macrocephaly, respectively [59], while the reciprocal diametric changes in head size were reported for 16p11.2 rearrangements [60],[61]. These observations and the associations of these genomic disorders with autism spectrum disorder (ASD) (1q21.1 duplication and 16p11.2 deletion) and schizophrenia (1q21.1 deletion and 16p11.2 duplication) [59],[61]–[72] lend support to the hypothesis that these conditions are at different ends of a spectrum related to evolution of the social brain [73],[74]. SMS and PTLS, like 1q21.1 and 16p11.2 rearrangements, are so-called genomic sister-disorders—disease mediated by duplications versus deletions of the same regions—with overlapping phenotypic traits (for a complete list, see [75]) in which conditions/phenotypes appeared to be linked to gene dosage. However, patients presenting ASD and 1q21.1 deletions or 16p11.2 duplications, as well as individuals with schizophrenia associated with 1q21.1 duplications or 16p11.2 deletions, were also reported ([61]–[65],[72]; reviewed in [74]), suggesting that some conditions might be due to altered gene(s) function(s) through both under- and overexpression. Alternatively, we can hypothesize that some phenotypes are not associated with a specific number of copies of a particular CNV but rather that the simple presence of a structural change at a given position of the human genome may cause perturbation in particular pathways regardless of gene dosage.

Murine genes mapping centromeric or telomeric to the SMS/PTLS rearrangement show analogous changes in expression. Specifically, a MMU11 gene over- or underexpressed in the SMS mouse model has more than 50% chance to be also over- or underexpressed in the PTLS mouse model, respectively. Remarkably, affected genes are mapping on the entirety of the chromosome and not only in proximity to the breakpoints. The uncoupling between the number of copies of the CNV genes and the phenotype, here the effect on expression of genes outside of the rearrangement, is further illustrated by the fact that we detect the same changes in expression in the compound heterozygote, i.e. a mouse model with a normal number of copies in a *cis* configuration (Figure 1). Concomitantly, this restoration of gene copy number within a structural change was shown not to rescue all phenotypic manifestations observed in the SMS and PTLS mice. Indeed some complex phenotypes such as activity, anxiety, and preference for social novelty were still present in these animals. These observations suggest a contribution of genomic structural changes to the final phenotypic outcome and experimentally document that simple gene dosage alone cannot account for these phenotypes. The non-concordant absence of compensation in *Df(11)17/Dp(11)17* mice (i.e. *Df(11)17/Dp(11)17* mice anxiety mimics the phenotype observed in the SMS model, while their preference for social novelty is similar to that of PTLS animals; Table 1) further uncovers the complexity resulting of CNV-related genomic alteration.

The activity levels measured in the open field test exemplify the interaction between gene dosage and final phenotypic outcome of a specific CNV. *Df(11)17/+* mice are hypoactive while *Dp(11)17/+* are hyperactive, hence the opposing phenotypes implicate gene dosage in the final outcome. Consistently, *Rai1* +/− heterozygote and *Rai1* transgenic mice were found to be hypo- and hyperactive in the open field, respectively [27],[76]. However, the compound heterozygote *Df(11)17/Dp(11)17* and *Dp(11)17/Rai-* mice [27] are also hyperactive in the open field, establishing that we are confronted with a complex phenotypic outcome. In conclusion, the presence of a CNV generates a phenotype through gene dosage imbalance and/or the presence of genomic structural changes. Further studies are warranted to resolve the underlying causes and assess the relevance of our findings beyond genetically engineered model and/or rare and highly penetrant CNVs.

Although we performed a broad battery of behavioral experiments and studied the gene expression profile in five tissues to address different aspects of SMS/PTLS phenotypes, there are still other facets that are yet to be studied. One of the most significant and consistent phenotypes displayed by almost all SMS patients is sleep disturbance, including early sleep onset and offset, repeated and prolonged nocturnal awakening, as well as excessive daytime sleepiness (“sleep attacks”). Sleep disturbance in SMS is accompanied by intrinsically inverted melatonin rhythms and is often claimed by patients and their families as one of the most challenging aspects of the SMS spectrum [33],[77],[78]. We suggest that with approaches similar to this study, by combining expression analyses in the suprachiasmatic nucleus (SCN) and performing circadian experiments of the SMS mouse models, valuable insights can be gained also for this important SMS phenotype.

Importantly, our results suggest that the pathways through which CNVs (including both deletions and duplications) result in complex traits, particularly those involving the CNS, might include not only alteration of the expression of genes included in the rearranged interval but also the subtle modification of the regulation of gene(s) mapping to the rest of the rearranged chromosome. These changes in expression levels might be triggered by a position effect, modification of the chromatin structure, perturbation of chromatin loops, disruption of long transcript structure, reflection of a regulatory interaction between chromosome homologues (e.g. transvection), and/or repositioning within the nucleus of a genomic region (e.g., in [79]–[83]; reviewed in [5]). Consistently, a balanced translocation was shown to significantly modify transcriptome profiles [84]. The results presented here also suggest that the chromosome and its gene collection are not randomly devised. The location and order are maintained possibly in relation to a higher level genomic organization required for proper regulation.

The potential unidirectionality of the long-range effects of CNVs on gene expression and phenotypic outcome independent of copy number change that has been uncovered in this report poses an important challenge in appreciating the contribution of this class of variation to phenotypic features. To include this variable in genome-wide [85] as well as in eQTL association studies [10], it might be necessary to combine all rearrangements that differ from normality regardless of their directionality.

## Materials and Methods

The materials and methods used for this report can be accessed online (Text S1).

## Supporting Information

Figure S1 **Neuromotor dysfunction in** ***Df(11)17/+*** **mice is gene dosage dependent.** (A) The total number of falls in the dowel test for each of the genotypes is depicted. (B) The average time in seconds that mice from each genotype could be hanging from a wire is shown. For each genotype the number of tested mice was: *N* = 14 for *Dp(11)17/+*, *N* = 9 for *Df(11)17/+*, *N* = 14 for +/+, and *N* = 12 for *Df(11)17/Dp(11)17* mice. The performance in the rotating rod is normal in mice with the correct gene dosage within this specific genomic interval. (C) Average time on top of the rotating rod for *Dp(11)17/+* (light grey squares) and +/+ (white squares), (D) *Df(11)17/+* (dark grey squares) and +/+ (white squares), and (E) *Df(11)17/Dp(11)17* (black squares) and +/+ (white squares) are represented. For each genotype the number of mice tested in the rotating rod was: *N* = 14 for *Dp(11)17/+*, *N* = 6 for *Df(11)17/+*, *N* = 12 for +/+, and *N* = 8 for *Df(11)17/Dp(11)17* mice. The mean ± S.E.M. values are presented. Asterisk denotes significantly different (* *p*<0.05).
(0.62 MB TIF)

Figure S2 **Craniofacial abnormalities are dependent on gene CNV within this genomic interval.** (A) *Dp(11)17/+*, (B) *Df(11)17/+*, (C) +/+, and (D) *Df(11)17/Dp(11)17* mice facial and skull pictures are shown. Note the position of the snout and the broader distance between the eyes (hypertelorism) for the *Df(11)17/+* mouse compared with the other mice. The shorter distance between the eyes and the nose can also be visualized in the *Df(11)17/+* mice. (E–L) Skeletal preparations of *Dp(11)17/+* (E, I), *Df(11)17/+* (F, J), wild type (G, K), and *Df(11)17/Dp(11)17* (H, L) skulls of 3-mo male animals are shown for comparison. The shape of the nasal bone of the *Df(11)17/+* mice is shown with an arrow (J). This phenotype is completely rescued with the addition of an extra copy of the genes that are deleted (*Df(11)17/Dp(11)17* animals) (L). (K) The different landmarks pictured in (C, I) were used to objectively measure the distances between them. Cranial landmarks (letter label) are as follows: b: nasal; a and c: anterior notch on frontal process lateral to intraorbital fissure; d: intersection of parietal and intraparietal bones; e: intersection of the interparietal and occipital bones at the midline; f: bregma; g: intersection of maxilla and sphenoid on inferior alveolar ridge. The relative distances (in centimeters; see Materials and Methods) were used for the statistical analysis, and the averages of the distances are shown in (M). The asterisk denotes significant differences (*p*<0.05). An *N* = 3 was utilized for each genotype.
(2.91 MB TIF)

Figure S3 **Weight differences are recovered with the correct (i.e., diploid 2n) gene copy number within this genomic interval.** (A) Total body weight in grams, and (B) abdominal fat weight in grams are depicted for *Dp(11)17/+* (*N* = 8) light grey columns, *Df(11)17/+* (*N* = 7) dark grey columns, +/+ (*N* = 8) white columns, and *Df(11)17/Dp(11)17* (*N* = 8) black columns. The mean ± S.E.M. values are presented. The asterisk denotes significant differences (*p*<0.05).
(0.43 MB TIF)

Figure S4 **The results for the first and second round of the tube test for social dominance are depicted as the percentage of winning for each genotype for (A) +/+ (white columns) versus** ***Dp(11)17/+*** **(light grey columns) (** ***N*** **= 10) mice, (B) +/+ (white columns) versus** ***Df(11)17/+*** **mice (dark grey columns) (** ***N*** **= 10), and (C) +/+ (white columns) versus** ***Df(11)17/Dp(11)17*** **(black columns) (** ***N*** **= 10) mice.**
(0.29 MB TIF)

Figure S5 **Relative expression levels measured by quantitative PCR.** Ratio of aneuploid/euploid normalized relative expression levels measured by quantitative PCR in male cerebellum (A), female hippocampus (B), and female lung (C). The comparisons between *Df(11)17/+* (SMS model, 1n) and *+/+* (2n) and *Dp(11)17/+* (PTLS model, 3n) and *+/+* (2n) are shown with burgundy squares and blue triangles, respectively (see Figure 1 for a schematic representation of the mouse 11 B2 region of the different mouse models). The assayed genes are ordered according to their mapping order on MMU11. Note that the SMS/PTLS engineered region maps from *Cops3* to *Zfp179*. Genes and assays are presented in Table S2.
(0.66 MB TIF)

Figure S6 **Expression levels of flanking genes before and after recombination.** Comparison of relative expression levels measured by quantitative PCR in *Dp(11)17/+* and *+/+* littermates before and after recombination. The selected genes showed significant differences in expression between *Dp(11)17/+* and *+/+* animals in the microarray profiling experiments (see main text for details). They map to a 16 megabase (coordinates MMU11:76843886-92963733) interval that recombined from a 129S5/C57BL6/J heterozygous background to a C57BL6/J/C57BL6/J homozygous background between the 12^th^ and 17^th^ backcross in *Dp(11)17/+* model animals as schematically shown in (A). Amplification results obtained in kidney (B), testis (C), and cerebellum (D) for three different male individuals of each genotype and backcross are shown. Blue and green triangles denote *+/+* animals after 12^th^ and 17^th^ backcross, respectively, while red and black disks indicate *Dp(11)17/+* animals after 12^th^ and 17^th^ backcross, respectively. Genes and assays are presented in Table S3.
(0.38 MB PDF)

Figure S7 **Cumulative distribution of the probesets showing a differential expression between C57BL6/J and 129S2 mice.** The 129 probesets were removed to create the Most-diff-restricted dataset (see main text for details).
(0.50 MB TIF)

Figure S8 **Differentially expressed genes in SMS and PTLS mouse models.** Heatmap of the changes in expression levels of the 36 Most-diff-restricted transcripts mapping to the SMS/PTLS rearranged interval (A) and the remainder of mouse chromosome 11 (59 transcripts) (B) measured in *Df(11)17/+* (d), *Dp(11)17/+*, and *Df(11)17/Dp(11)17* (dD) mice as compared to +/+ individuals in cerebellum (C), heart (H), kidney (K), testis (T), and hippocampus (Hi). The arrowhead and asterisk denote *Cops3* and *Zfp179* transcripts, respectively. These transcripts were used as anchors in the strain engineering process, thus they are not present in the same number of copies than other SMS/PTLS genes in the mice models (see Figure 1 and text for details).
(1.52 MB TIF)

Figure S9 **Genes differentially expressed in SMS and PTLS mouse models map along the entire length of mouse chromosome 11.** Normalized relative expression of aneuploid/euploid in the vicinity of the SMS/PTLS region (A) or along the entirety of mouse chromosome 11 (B) for Most-diff-restricted dataset. The four top panels show measurements in four different tissues (C, cerebellum; H, heart; K, kidney; T, testis), while the bottom panel presents the merge of all data. The following comparisons are shown: *Df(11)17/+* (SMS model, 1n) to *+/+* (2n) with squares; *Dp(11)17/+* (PTLS model, 3n) to *+/+* (2n) with triangles; and *Df(11)17/Dp(11)17* (2n compound heterozygote) to *+/+* (2n) with disks (see Figure 1 for a schematic representation of the mouse 11 B2 region of the different mouse models). The genes, which show statistically significant changes in expression between aneuploid and euploid models, are depicted with colored signs. Chromosome 11 coordinates are shown below. The SMS/PTLS engineered region is highlighted in light purple. Relative gene density along the chromosome is indicated in the bottom panels with a black line. The region between the red dotted lines in (A) is enlarged in (B).
(4.12 MB PDF)

Figure S10 **Affected transcripts show no correlation between extent of expression changes and distance from the breakpoints.** For each affected transcripts (Most-diff-restricted dataset), we plotted the expression changes between aneuploid and euploid animals in function of their distance to the breakpoints (top panel: *Df(11)17/+* versus *+/+*; central panel: *Dp(11)1/+* versus *+/+*; and bottom panel: *Df(11)17/Dp(11)17* versus *+/+*). Data for each assessed tissue were merged and the correlation coefficient (r) was calculated.
(0.57 MB TIF)

Figure S11 **The affected transcripts are not highly expressed.** For each tissue and each expressed transcript, the F-test value is plotted against the expression level measured in wild type (*+/+* genotype) or the 2n compound heterozygote (*Df(11)17/Dp(11)17*). Red signs and curve denote the transcripts belonging to the most differentially expressed set and their corresponding Lowess curve.
(0.70 MB TIF)

Table S1 **The viability of the different genotypes in this inbreed genetic background is dependent on gene dosage.** Typical matings between animals *Dp(11)17/+* × +/+, *Df(11)17/+* × +/+ and *Df(11)17/+* × *Dp(11)17/+* mice (12^th^ backcross in C57BL/6-*Tyr^c-Brd^* genetic background). The total numbers of mice born from each mating type is indicated, plus the resulting *n* of each genotype. The % of mice born/% expected for each genotype is shown. The * denotes significantly different from the expected Mendelian ratio. Gene copy number within this genomic interval is indicated in brackets for each genotype.
(0.03 MB DOC)

Table S2 **Genes and quantitative PCR assays to validate microarrays experiments.**
(0.05 MB XLS)

Table S3 **Genes and quantitative PCR assays before and after recombination in three tissues.**
(0.06 MB DOC)

Table S4 **Transcripts differentially expressed in the hippocampus of** ***Df(11)17/Dp(11)17*** **mouse.**
(0.08 MB XLS)

Table S5 **Transcripts differentially expressed in the cerebellum of** ***Df(11)17/Dp(11)17*** **mouse.**
(0.08 MB XLS)

Table S6 **Genes with abnormal expression in the compound heterozygous mice that can putatively explain the phenotypes found in** ***Df(11)17/Dp(11)17*** **.**
(0.05 MB DOC)

Text S1 **Online supplementary text and online supplementary materials and methods.**
(0.20 MB DOC)

## Abbreviations

ASD: autism spectrum disorder
CNV: Copy number variation
GEO: Gene Expression Omnibus
MMU11: *Mus musculus* chromosome 11
PCR: Polymerase Chain Reaction
PTLS: Potocki-Lupski syndrome
*RAI1*: Retinoic Acid Induced gene 1
SCN: suprachiasmatic nucleus
SD: Standard Deviation
SMS: Smith-Magenis Syndrome
