# Supplementary figures and images for: Phenotypic Consequences of Copy Number Variation: Insights from Smith-Magenis and Potocki-Lupski Syndrome Mouse Models
Source: PLoS Biol. 2010 Nov 23;8(11):e1000543. doi: 10.1371/journal.pbio.1000543 (PMC2990707; doi:10.1371/journal.pbio.1000543)

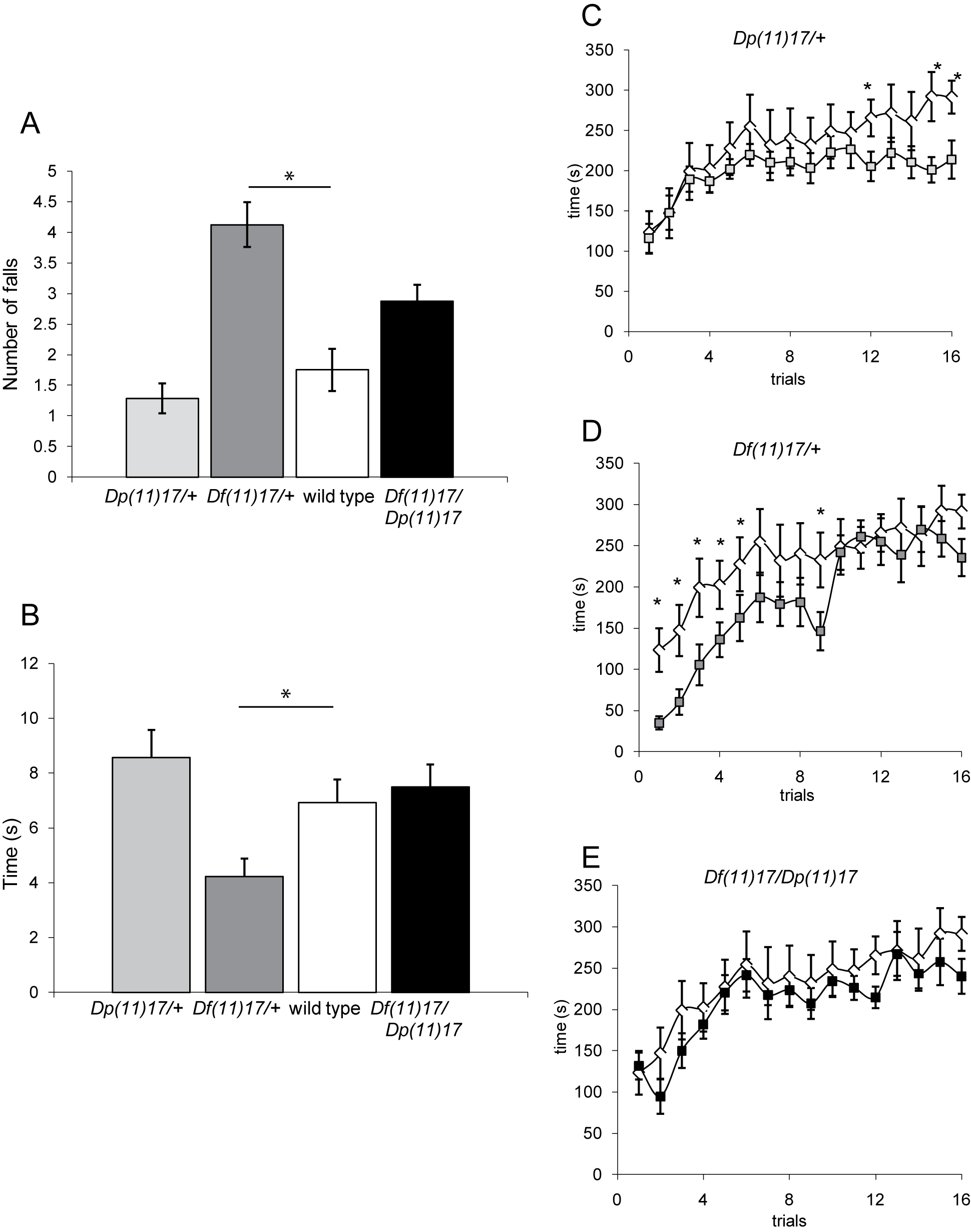

Supplement: Figure S1 — Neuromotor dysfunction in Df(11)17/+ mice is gene dosage dependent. (A) The total number of falls in the dowel test for each of the genotypes is depicted. (B) The average time in seconds that mice from each genotype could be hanging from a wire is shown. For each genotype the number of tested mice was: N = 14 for Dp(11)17/+, N = 9 for Df(11)17/+, N = 14 for +/+, and N = 12 for Df(11)17/Dp(11)17 mice. The performance in the rotating rod is normal in mice with the correct gene dosage within this specific genomic interval. (C) Average time on top of the rotating rod for Dp(11)17/+ (light grey squares) and +/+ (white squares), (D) Df(11)17/+ (dark grey squares) and +/+ (white squares), and (E) Df(11)17/Dp(11)17 (black squares) and +/+ (white squares) are represented. For each genotype the number of mice tested in the rotating rod was: N = 14 for Dp(11)17/+, N = 6 for Df(11)17/+, N = 12 for +/+, and N = 8 for Df(11)17/Dp(11)17 mice. The mean ± S.E.M. values are presented. Asterisk denotes significantly different (* p<0.05). (0.62 MB TIF) [file pbio.1000543.s001.tif]

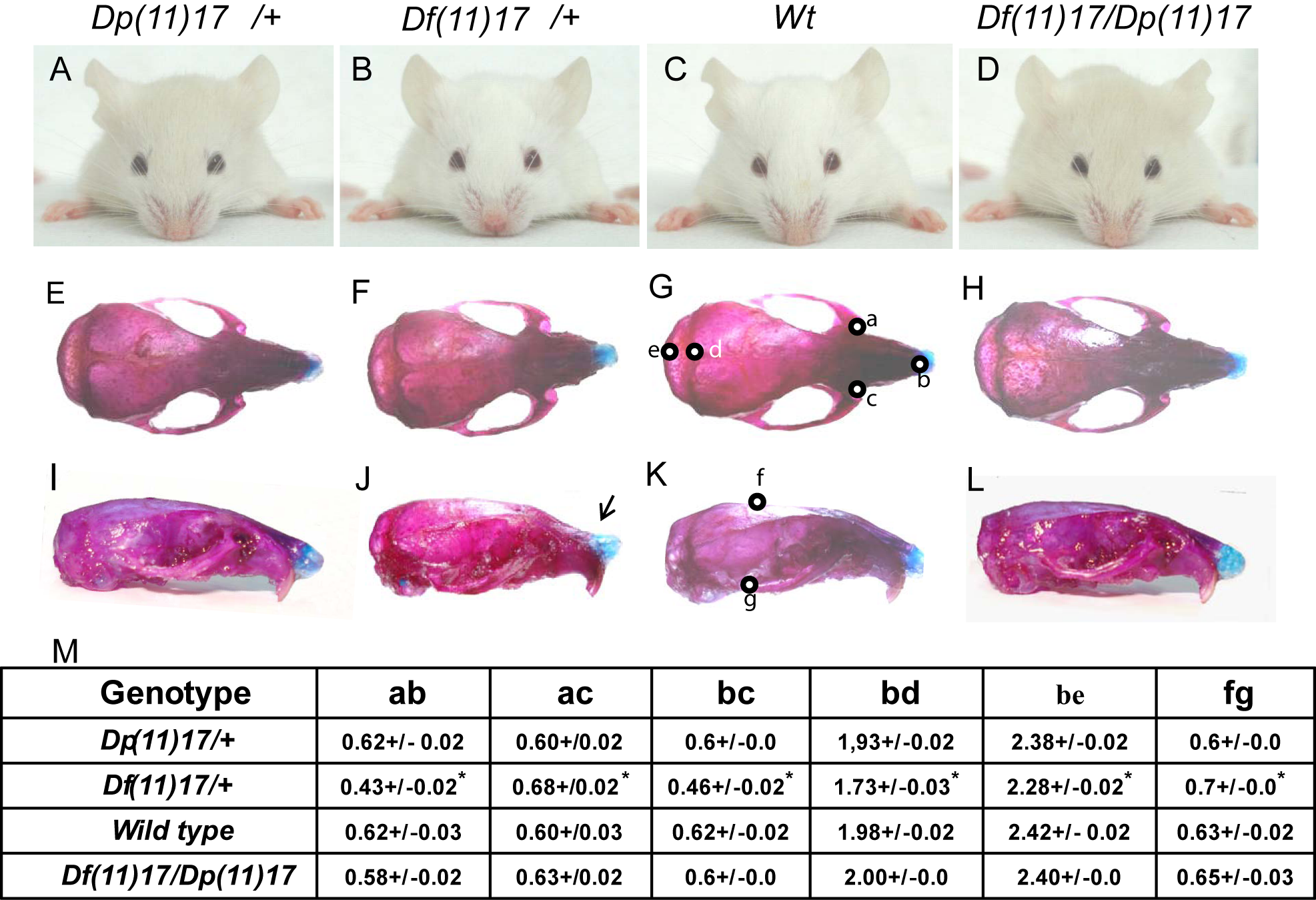

Supplement: Figure S2 — Craniofacial abnormalities are dependent on gene CNV within this genomic interval. (A) Dp(11)17/+, (B) Df(11)17/+, (C) +/+, and (D) Df(11)17/Dp(11)17 mice facial and skull pictures are shown. Note the position of the snout and the broader distance between the eyes (hypertelorism) for the Df(11)17/+ mouse compared with the other mice. The shorter distance between the eyes and the nose can also be visualized in the Df(11)17/+ mice. (E–L) Skeletal preparations of Dp(11)17/+ (E, I), Df(11)17/+ (F, J), wild type (G, K), and Df(11)17/Dp(11)17 (H, L) skulls of 3-mo male animals are shown for comparison. The shape of the nasal bone of the Df(11)17/+ mice is shown with an arrow (J). This phenotype is completely rescued with the addition of an extra copy of the genes that are deleted (Df(11)17/Dp(11)17 animals) (L). (K) The different landmarks pictured in (C, I) were used to objectively measure the distances between them. Cranial landmarks (letter label) are as follows: b: nasal; a and c: anterior notch on frontal process lateral to intraorbital fissure; d: intersection of parietal and intraparietal bones; e: intersection of the interparietal and occipital bones at the midline; f: bregma; g: intersection of maxilla and sphenoid on inferior alveolar ridge. The relative distances (in centimeters; see Materials and Methods) were used for the statistical analysis, and the averages of the distances are shown in (M). The asterisk denotes significant differences (p<0.05). An N = 3 was utilized for each genotype. (2.91 MB TIF) [file pbio.1000543.s002.tif]

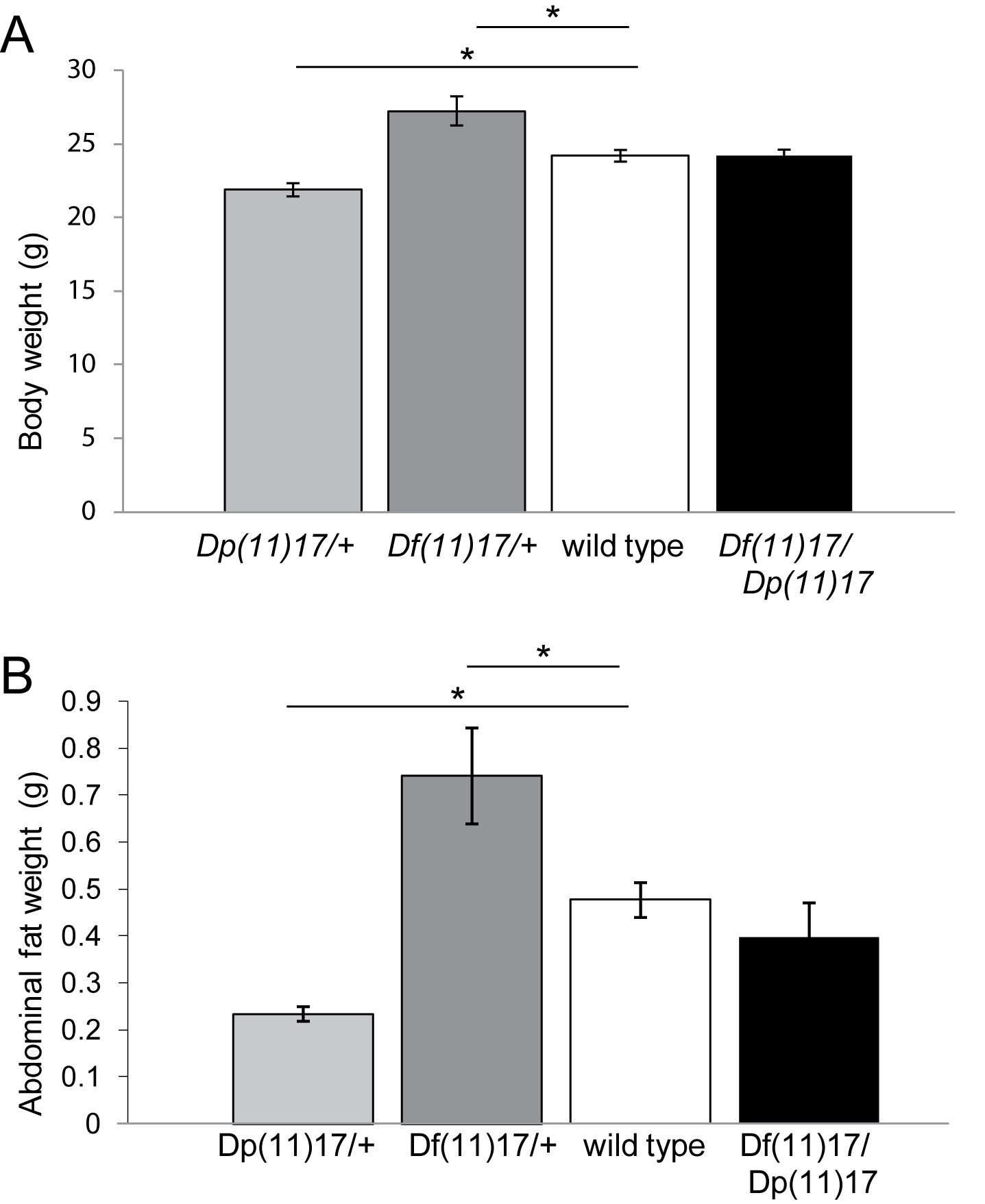

Supplement: Figure S3 — Weight differences are recovered with the correct (i.e., diploid 2n) gene copy number within this genomic interval. (A) Total body weight in grams, and (B) abdominal fat weight in grams are depicted for Dp(11)17/+ (N = 8) light grey columns, Df(11)17/+ (N = 7) dark grey columns, +/+ (N = 8) white columns, and Df(11)17/Dp(11)17 (N = 8) black columns. The mean ± S.E.M. values are presented. The asterisk denotes significant differences (p<0.05). (0.43 MB TIF) [file pbio.1000543.s003.tif]

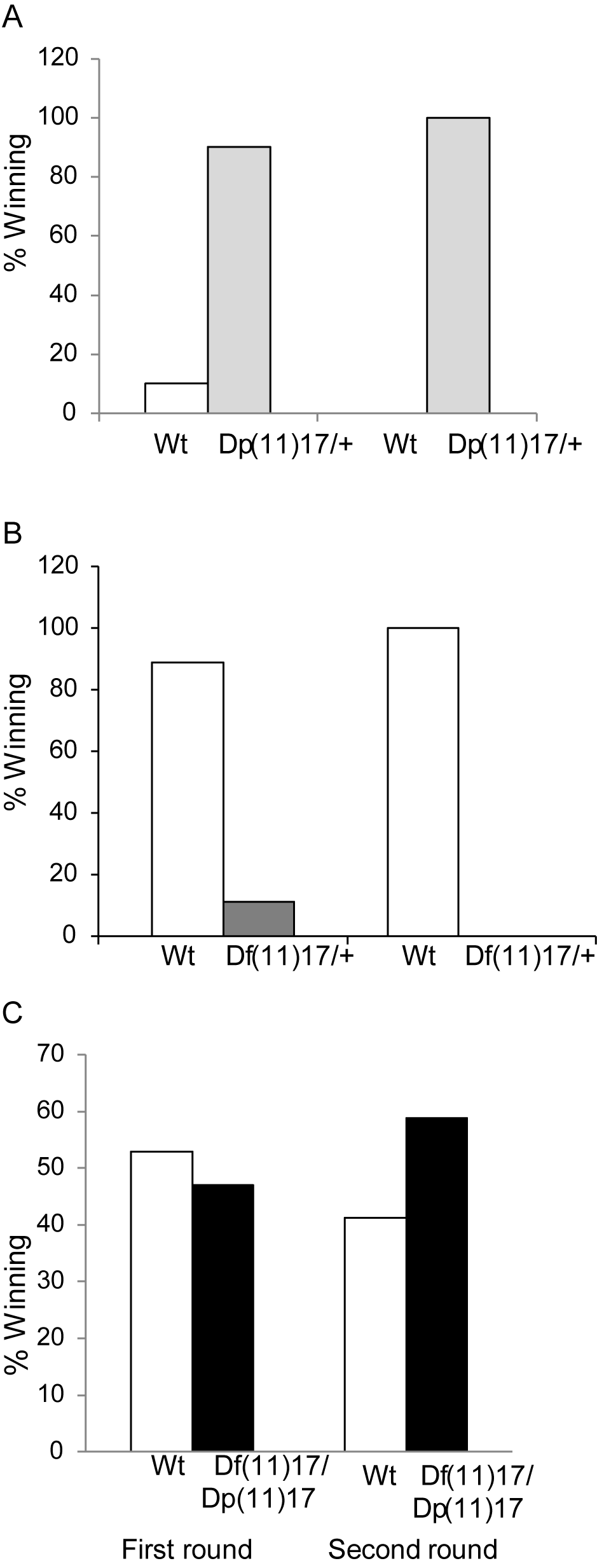

Supplement: Figure S4 — The results for the first and second round of the tube test for social dominance are depicted as the percentage of winning for each genotype for (A) +/+ (white columns) versus Dp(11)17/+ (light grey columns) ( N = 10) mice, (B) +/+ (white columns) versus Df(11)17/+ mice (dark grey columns) ( N = 10), and (C) +/+ (white columns) versus Df(11)17/Dp(11)17 (black columns) ( N = 10) mice. (0.29 MB TIF) [file pbio.1000543.s004.tif]

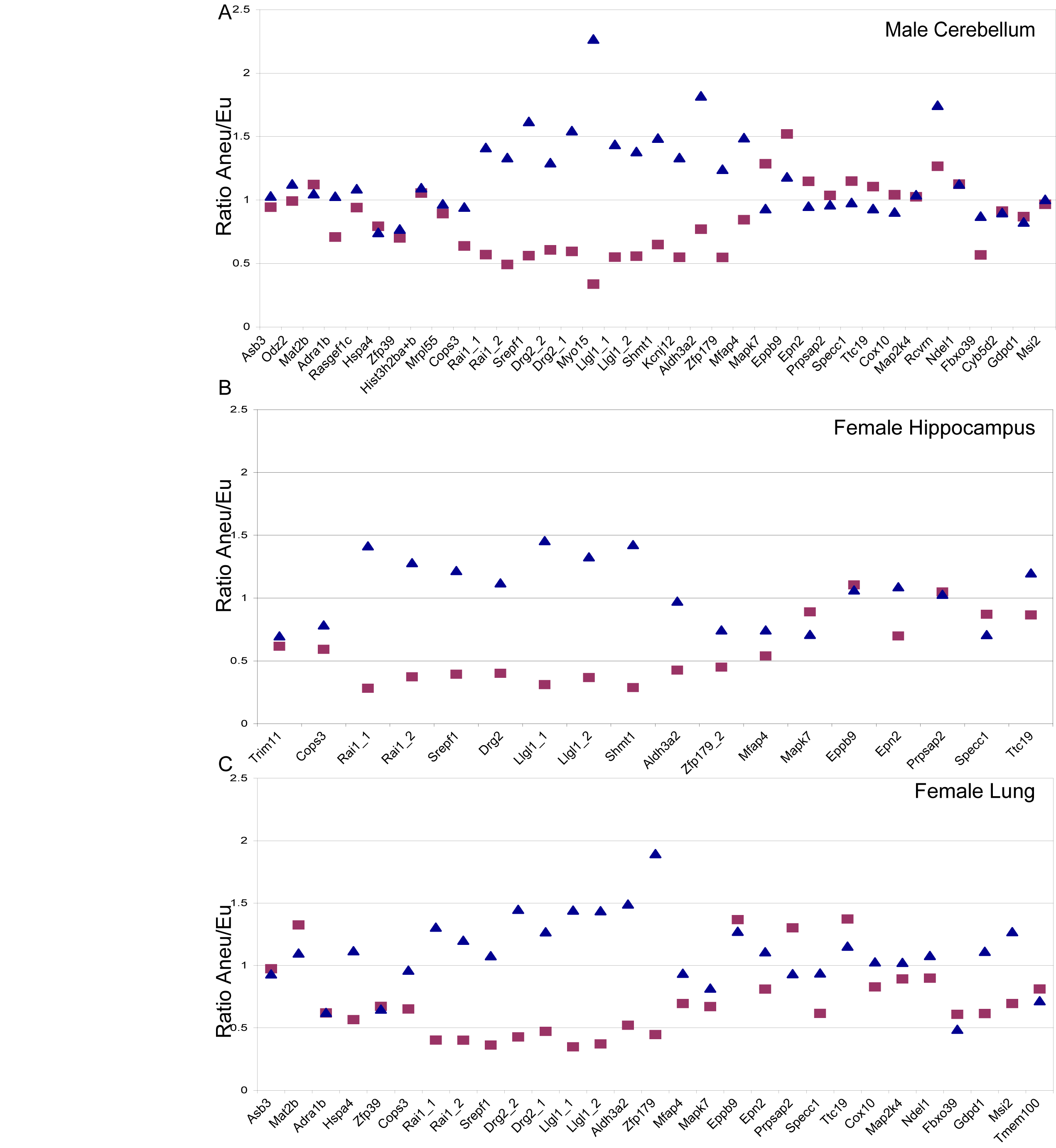

Supplement: Figure S5 — Relative expression levels measured by quantitative PCR. Ratio of aneuploid/euploid normalized relative expression levels measured by quantitative PCR in male cerebellum (A), female hippocampus (B), and female lung (C). The comparisons between Df(11)17/+ (SMS model, 1n) and +/+ (2n) and Dp(11)17/+ (PTLS model, 3n) and +/+ (2n) are shown with burgundy squares and blue triangles, respectively (see Figure 1 for a schematic representation of the mouse 11 B2 region of the different mouse models). The assayed genes are ordered according to their mapping order on MMU11. Note that the SMS/PTLS engineered region maps from Cops3 to Zfp179. Genes and assays are presented in Table S2. (0.66 MB TIF) [file pbio.1000543.s005.tif]

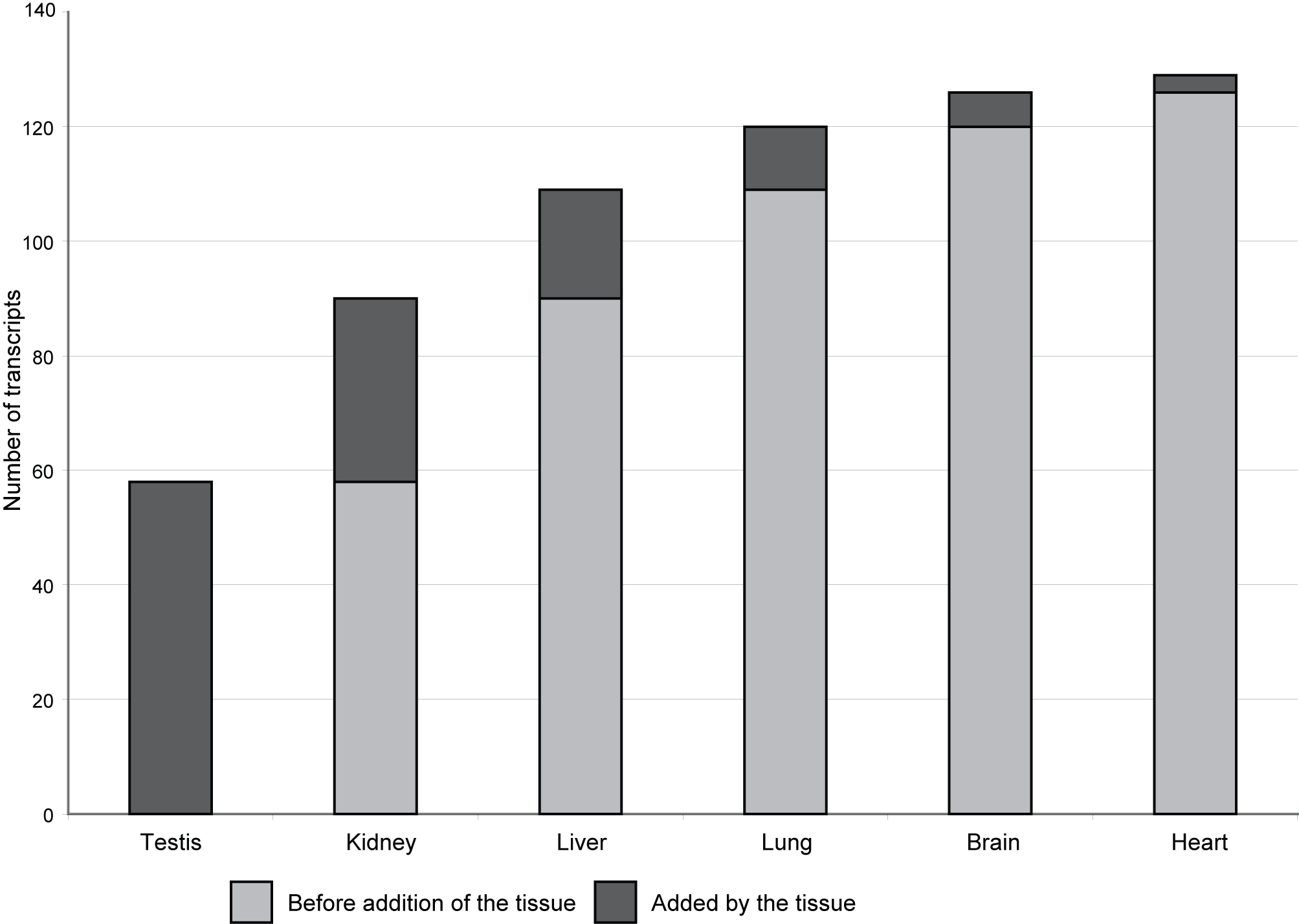

Supplement: Figure S7 — Cumulative distribution of the probesets showing a differential expression between C57BL6/J and 129S2 mice. The 129 probesets were removed to create the Most-diff-restricted dataset (see main text for details). (0.50 MB TIF) [file pbio.1000543.s007.tif]

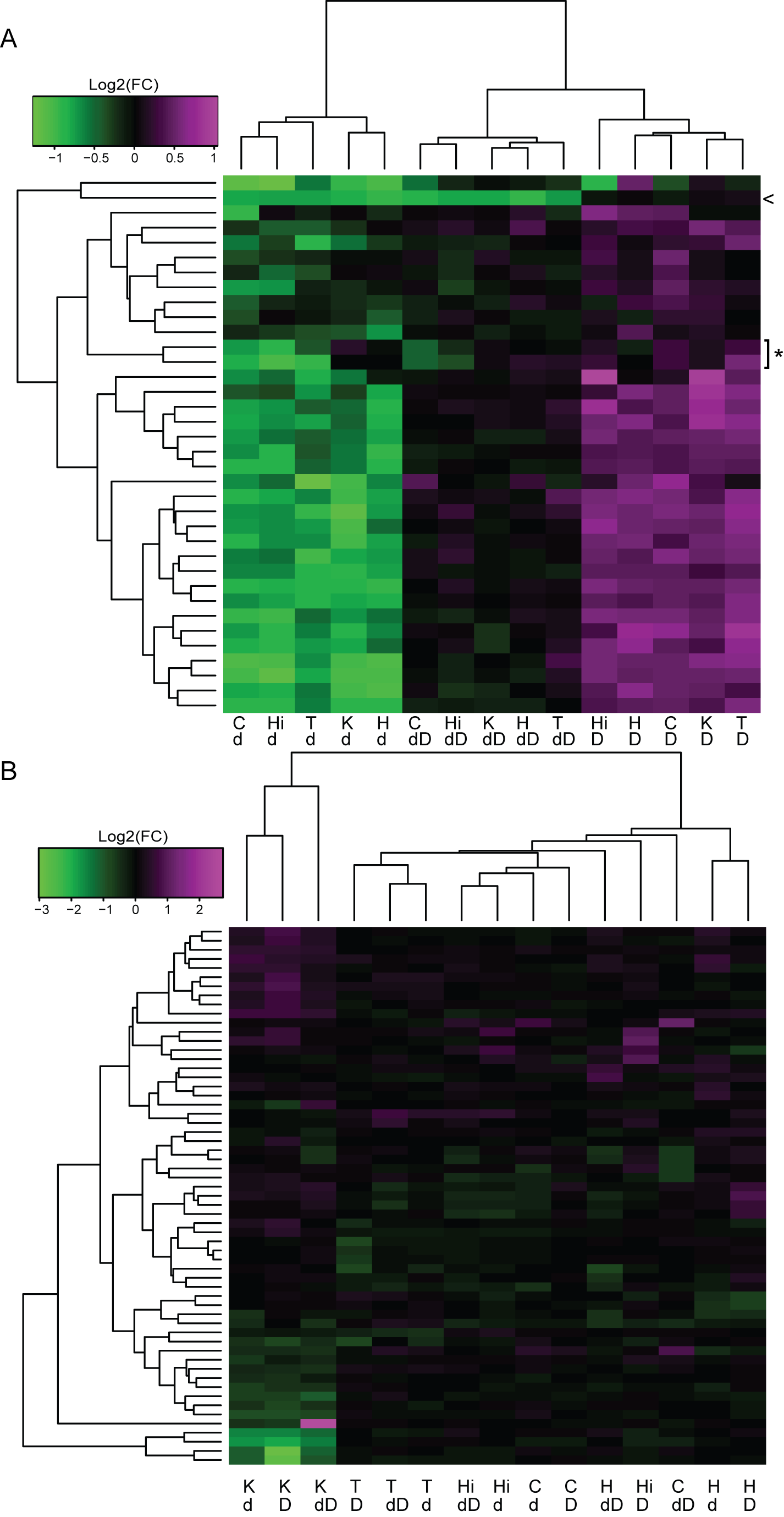

Supplement: Figure S8 — Differentially expressed genes in SMS and PTLS mouse models. Heatmap of the changes in expression levels of the 36 Most-diff-restricted transcripts mapping to the SMS/PTLS rearranged interval (A) and the remainder of mouse chromosome 11 (59 transcripts) (B) measured in Df(11)17/+ (d), Dp(11)17/+, and Df(11)17/Dp(11)17 (dD) mice as compared to +/+ individuals in cerebellum (C), heart (H), kidney (K), testis (T), and hippocampus (Hi). The arrowhead and asterisk denote Cops3 and Zfp179 transcripts, respectively. These transcripts were used as anchors in the strain engineering process, thus they are not present in the same number of copies than other SMS/PTLS genes in the mice models (see Figure 1 and text for details). (1.52 MB TIF) [file pbio.1000543.s008.tif]

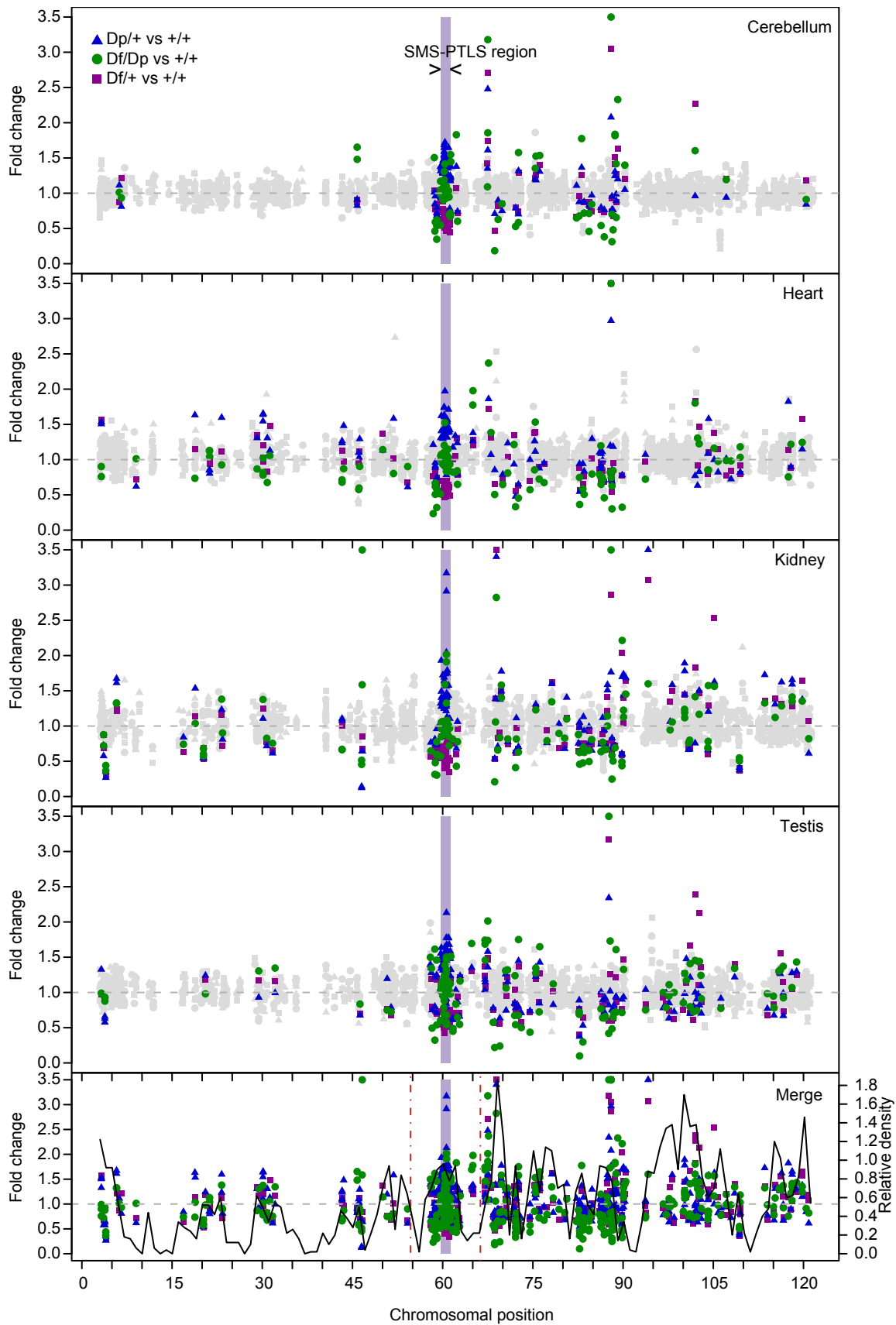

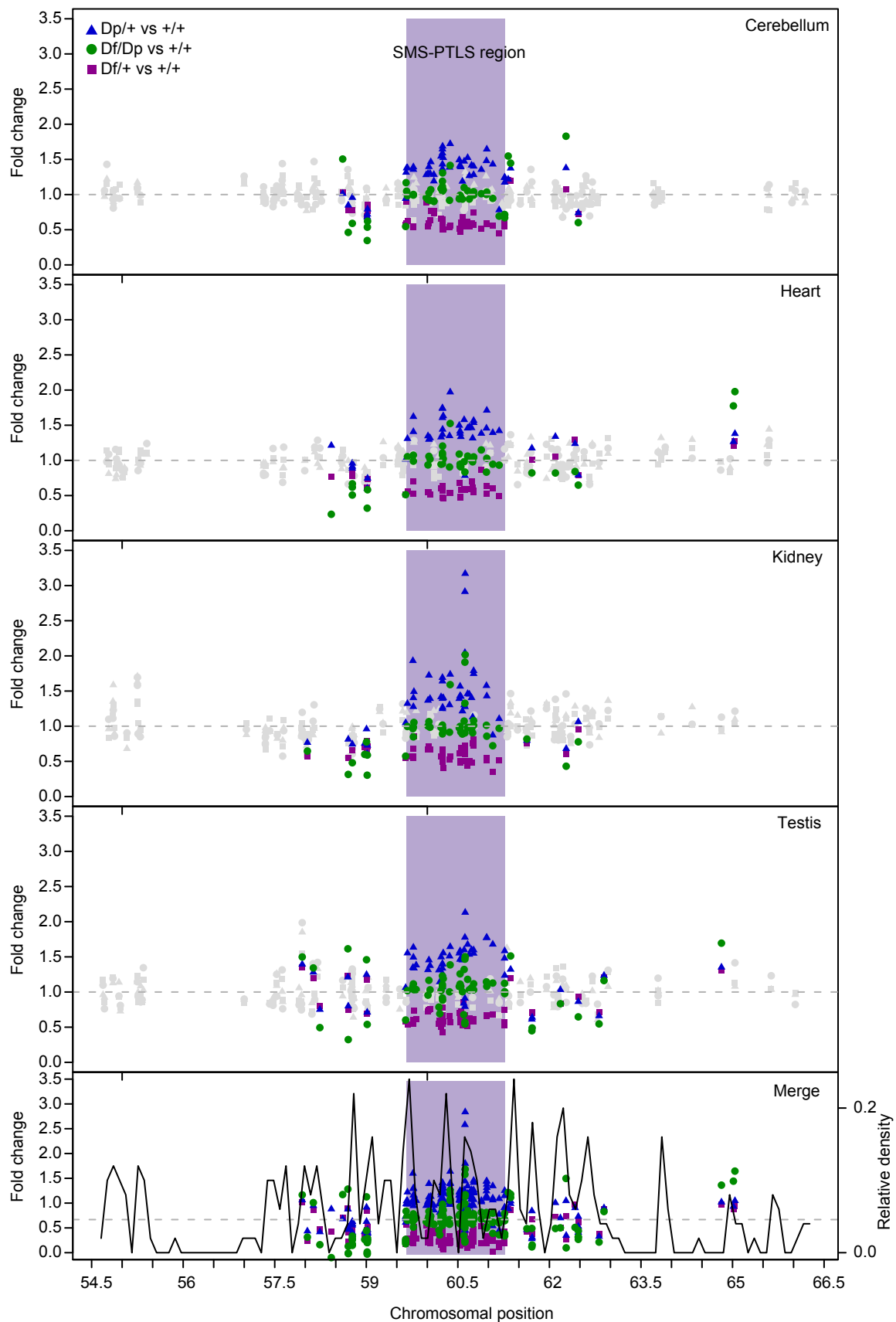

Supplement: Figure S9 — Genes differentially expressed in SMS and PTLS mouse models map along the entire length of mouse chromosome 11. Normalized relative expression of aneuploid/euploid in the vicinity of the SMS/PTLS region (A) or along the entirety of mouse chromosome 11 (B) for Most-diff-restricted dataset. The four top panels show measurements in four different tissues (C, cerebellum; H, heart; K, kidney; T, testis), while the bottom panel presents the merge of all data. The following comparisons are shown: Df(11)17/+ (SMS model, 1n) to +/+ (2n) with squares; Dp(11)17/+ (PTLS model, 3n) to +/+ (2n) with triangles; and Df(11)17/Dp(11)17 (2n compound heterozygote) to +/+ (2n) with disks (see Figure 1 for a schematic representation of the mouse 11 B2 region of the different mouse models). The genes, which show statistically significant changes in expression between aneuploid and euploid models, are depicted with colored signs. Chromosome 11 coordinates are shown below. The SMS/PTLS engineered region is highlighted in light purple. Relative gene density along the chromosome is indicated in the bottom panels with a black line. The region between the red dotted lines in (A) is enlarged in (B). (4.12 MB PDF) [file pbio.1000543.s009.pdf]

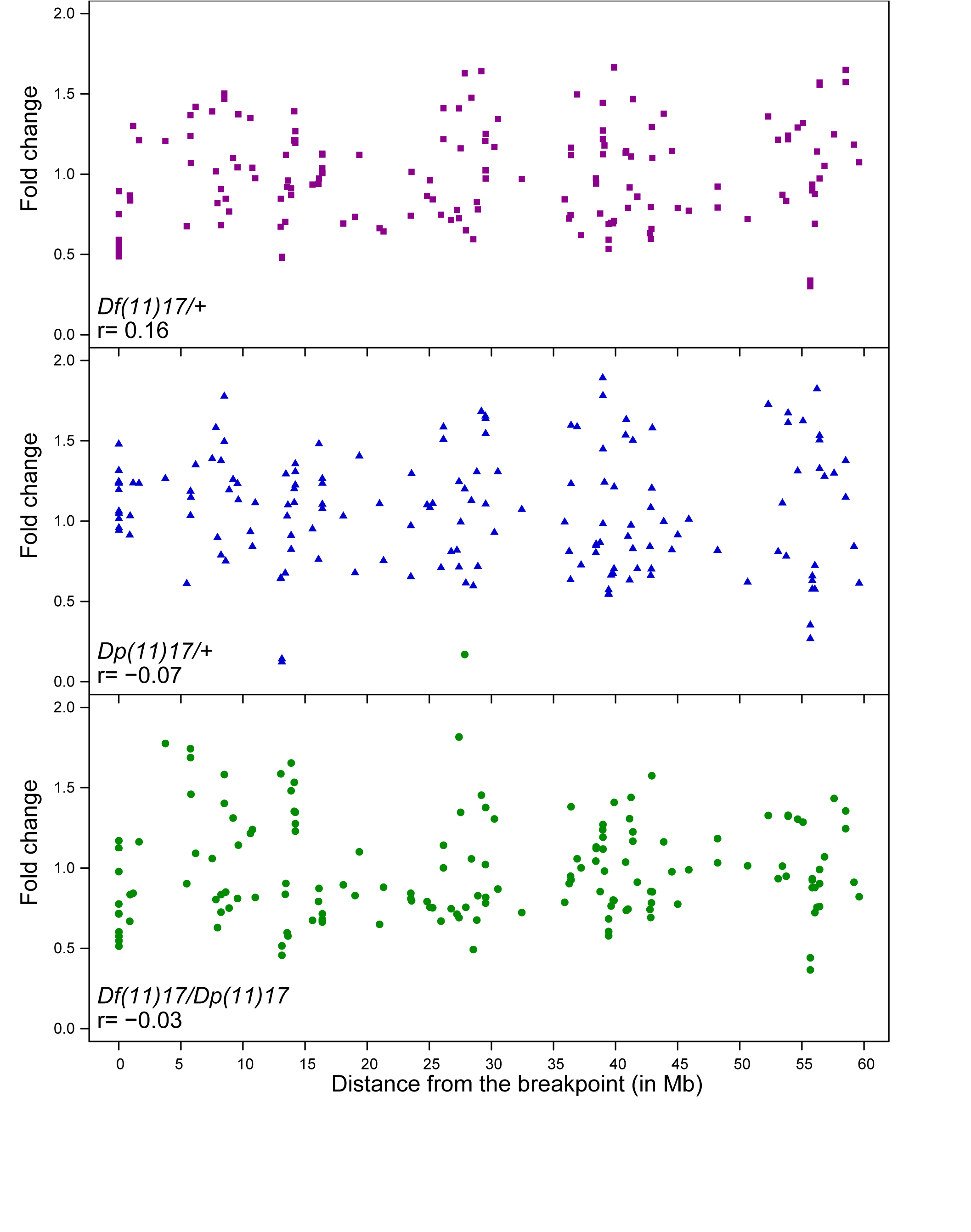

Supplement: Figure S10 — Affected transcripts show no correlation between extent of expression changes and distance from the breakpoints. For each affected transcripts (Most-diff-restricted dataset), we plotted the expression changes between aneuploid and euploid animals in function of their distance to the breakpoints (top panel: Df(11)17/+ versus +/+; central panel: Dp(11)1/+ versus +/+; and bottom panel: Df(11)17/Dp(11)17 versus +/+). Data for each assessed tissue were merged and the correlation coefficient (r) was calculated. (0.57 MB TIF) [file pbio.1000543.s010.tif]

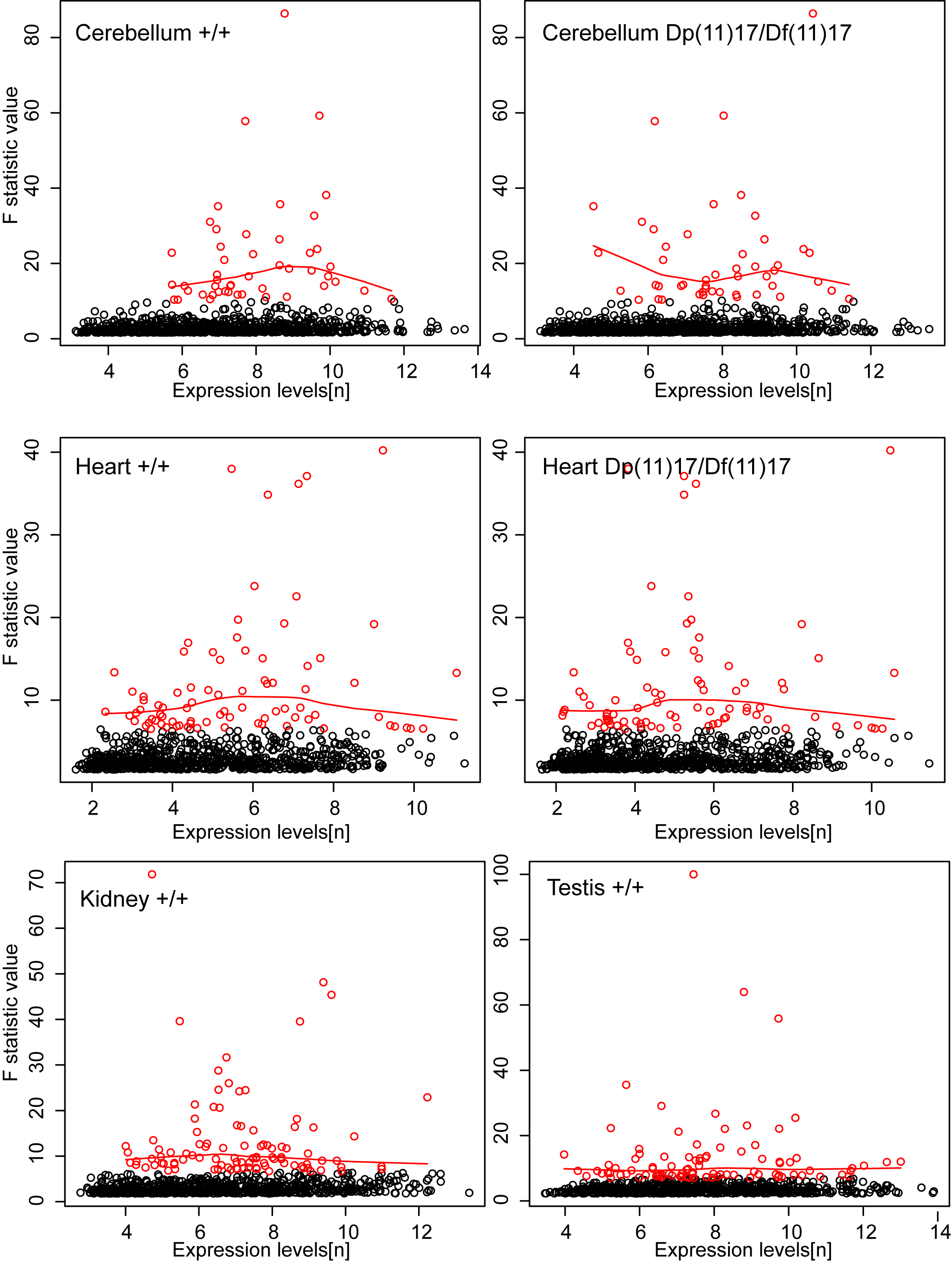

Supplement: Figure S11 — The affected transcripts are not highly expressed. For each tissue and each expressed transcript, the F-test value is plotted against the expression level measured in wild type (+/+ genotype) or the 2n compound heterozygote (Df(11)17/Dp(11)17). Red signs and curve denote the transcripts belonging to the most differentially expressed set and their corresponding Lowess curve. (0.70 MB TIF) [file pbio.1000543.s011.tif]
