# Supplementary material for: Phenotypic Consequences of Copy Number Variation: Insights from Smith-Magenis and Potocki-Lupski Syndrome Mouse Models
Source: PLoS Biol. 2010 Nov 23;8(11):e1000543. doi: 10.1371/journal.pbio.1000543 (PMC2990707; doi:10.1371/journal.pbio.1000543)
Supplement: Figure S6 — Expression levels of flanking genes before and after recombination. Comparison of relative expression levels measured by quantitative PCR in Dp(11)17/+ and +/+ littermates before and after recombination. The selected genes showed significant differences in expression between Dp(11)17/+ and +/+ animals in the microarray profiling experiments (see main text for details). They map to a 16 megabase (coordinates MMU11:76843886-92963733) interval that recombined from a 129S5/C57BL6/J heterozygous background to a C57BL6/J/C57BL6/J homozygous background between the 12th and 17th backcross in Dp(11)17/+ model animals as schematically shown in (A). Amplification results obtained in kidney (B), testis (C), and cerebellum (D) for three different male individuals of each genotype and backcross are shown. Blue and green triangles denote +/+ animals after 12th and 17th backcross, respectively, while red and black disks indicate Dp(11)17/+ animals after 12th and 17th backcross, respectively. Genes and assays are presented in Table S3. (0.38 MB PDF) [file pbio.1000543.s006.pdf]

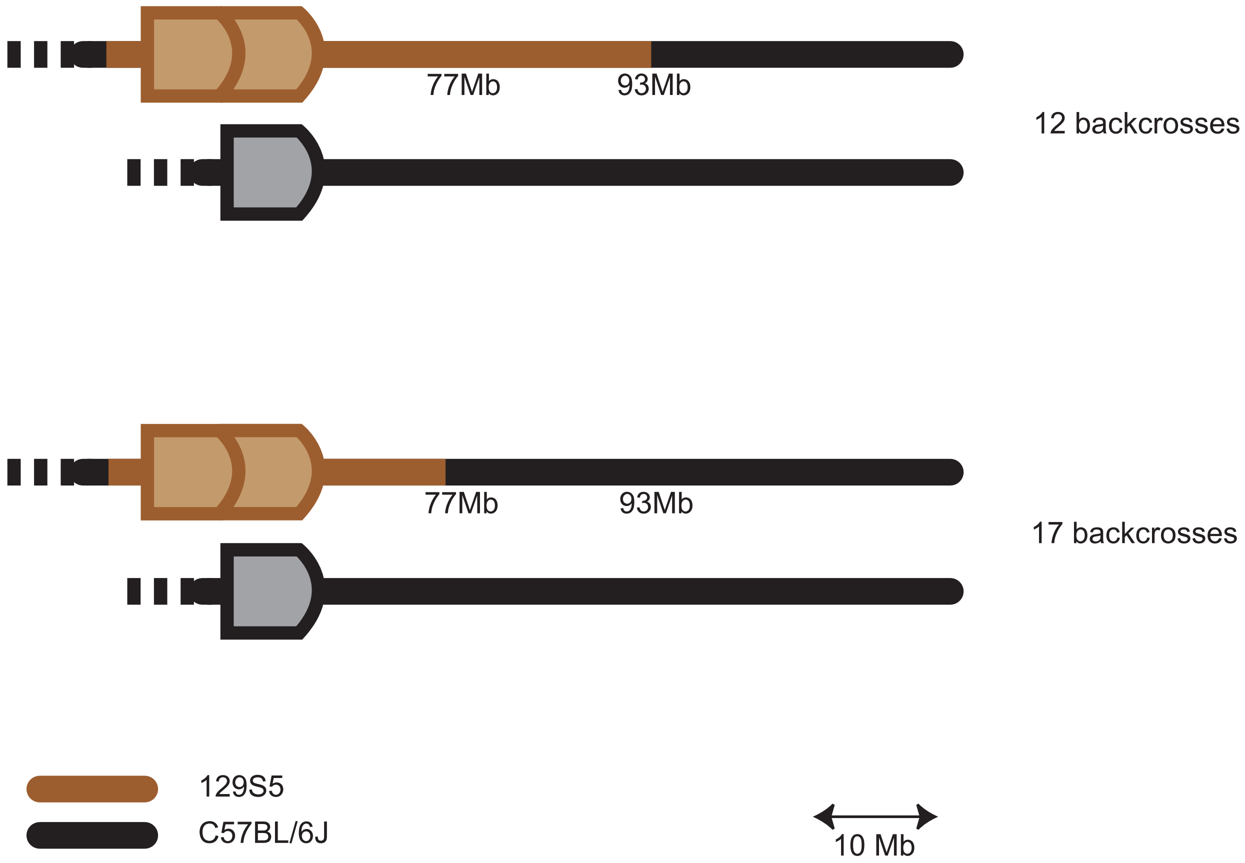

77Mb
93Mb
12 backcrosses
77Mb
93Mb
17 backcrosses
129S5
C57BL/6J
10 Mb

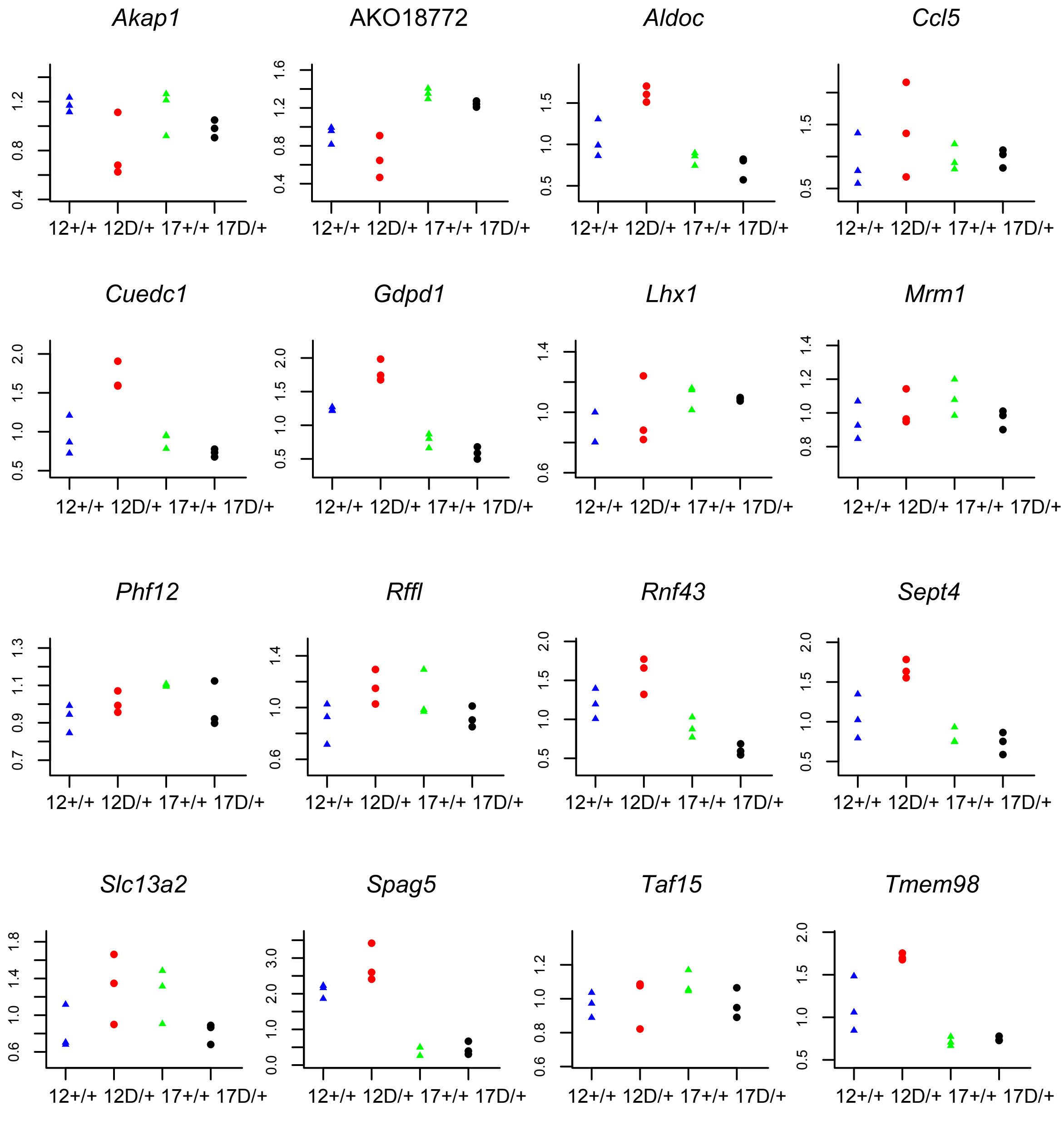
Akap1
AKO18772
Aldoc
Ccl5
Cuedc1
Gdpd1
Lhx1
Mrm1
Phf12
Rffl
Rnf43
Sept4
Slc13a2
Spag5
Taf15
Tmem98
12+/+ 12D/+ 17+/+ 17D/+

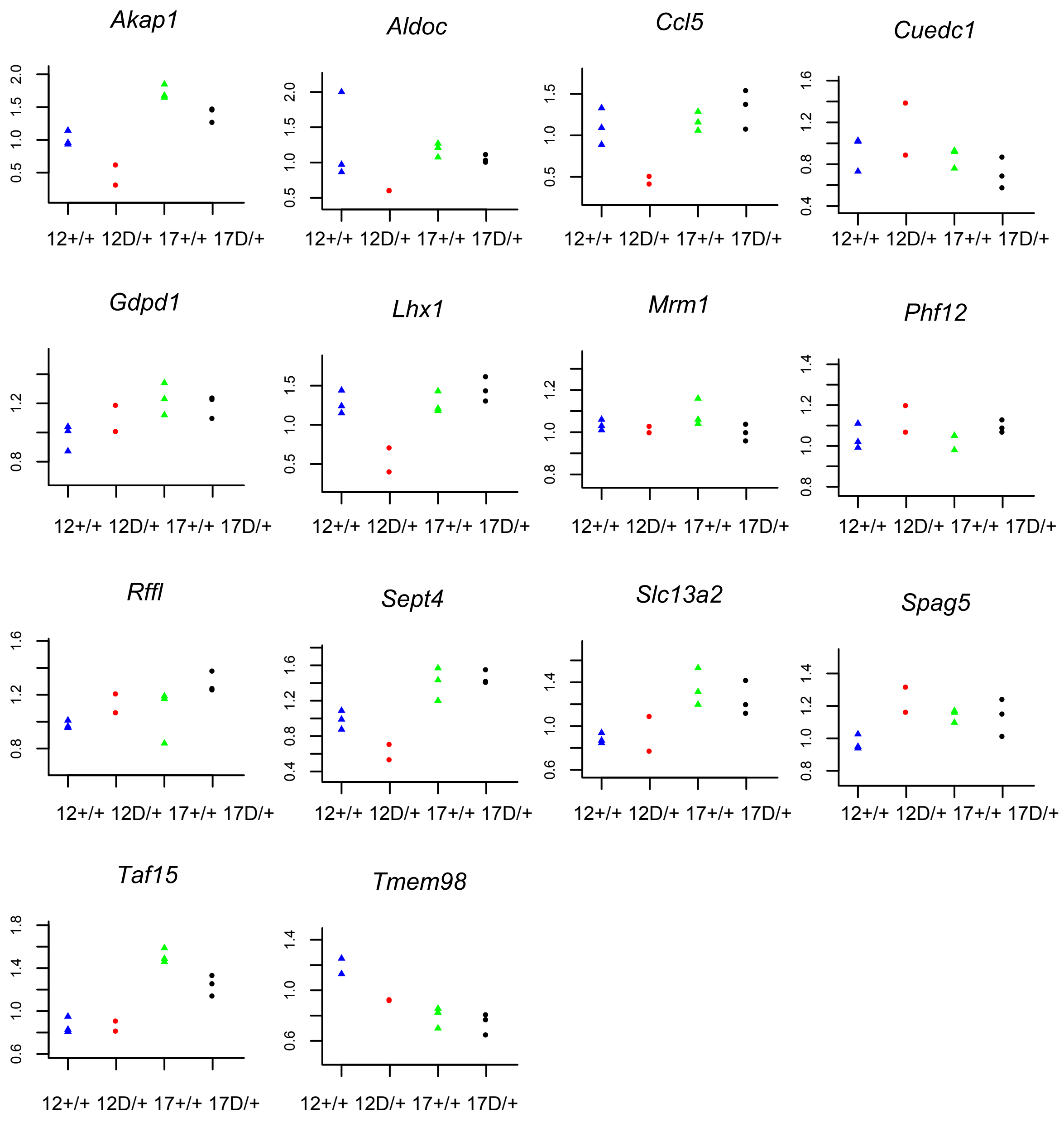

Akap1
2.0
1.5
1.0
0.5
12+/+ 12D/+ 17+/+ 17D/+
Aldoc
2.0
1.5
1.0
0.5
12+/+ 12D/+ 17+/+ 17D/+
Ccl5
1.5
1.0
0.5
12+/+ 12D/+ 17+/+ 17D/+
Cuedc1
1.6
1.2
0.8
0.4
12+/+ 12D/+ 17+/+ 17D/+
Gdpd1
1.2
0.8
12+/+ 12D/+ 17+/+ 17D/+
Lhx1
1.5
1.0
0.5
12+/+ 12D/+ 17+/+ 17D/+
Mrm1
1.2
1.0
0.8
12+/+ 12D/+ 17+/+ 17D/+
Phf12
1.4
1.2
1.0
0.8
12+/+ 12D/+ 17+/+ 17D/+
Rffl
1.6
1.2
0.8
12+/+ 12D/+ 17+/+ 17D/+
Sept4
1.6
1.2
0.8
0.4
12+/+ 12D/+ 17+/+ 17D/+
Slc13a2
1.4
1.0
0.6
12+/+ 12D/+ 17+/+ 17D/+
Spag5
1.4
1.2
1.0
0.8
12+/+ 12D/+ 17+/+ 17D/+
Taf15
1.8
1.4
1.0
0.6
12+/+ 12D/+ 17+/+ 17D/+
Tmem98
1.4
1.0
0.6
12+/+ 12D/+ 17+/+ 17D/+

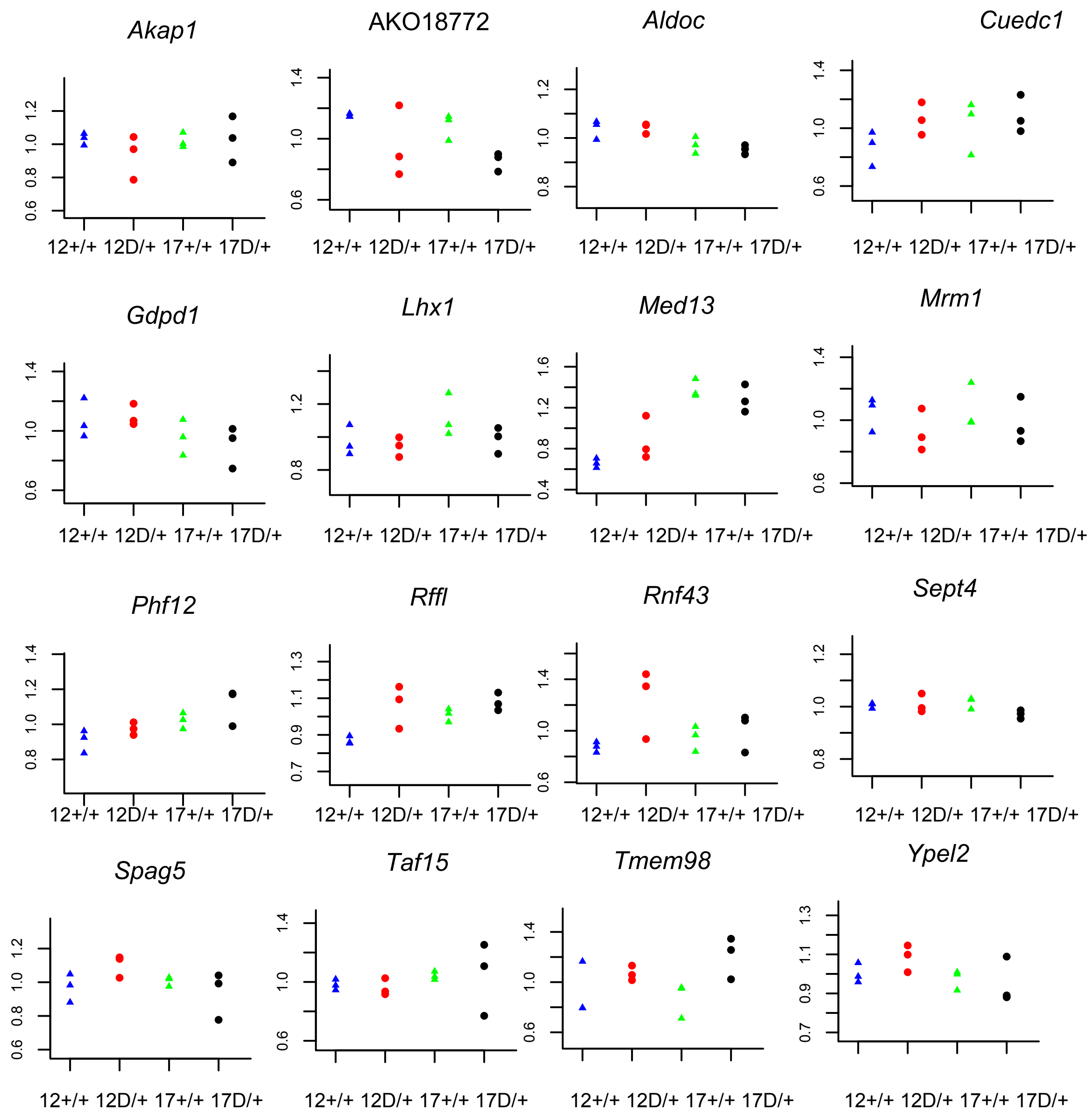

Akap1
AKO18772
Aldoc
Cuedc1
Gdpd1
Lhx1
Med13
Mrm1
Phf12
Rffl
Rnf43
Sept4
Spag5
Taf15
Tmem98
Ypel2
12+/+ 12D/+ 17+/+ 17D/+
