# Supplementary material for: Phenotypic Consequences of Copy Number Variation: Insights from Smith-Magenis and Potocki-Lupski Syndrome Mouse Models
Source: PLoS Biol. 2010 Nov 23;8(11):e1000543. doi: 10.1371/journal.pbio.1000543 (PMC2990707; doi:10.1371/journal.pbio.1000543)
Supplement: Table S1 — The viability of the different genotypes in this inbreed genetic background is dependent on gene dosage. Typical matings between animals Dp(11)17/+ × +/+, Df(11)17/+ × +/+ and Df(11)17/+ × Dp(11)17/+ mice (12th backcross in C57BL/6-Tyrc-Brd genetic background). The total numbers of mice born from each mating type is indicated, plus the resulting n of each genotype. The % of mice born/% expected for each genotype is shown. The * denotes significantly different from the expected Mendelian ratio. Gene copy number within this genomic interval is indicated in brackets for each genotype. (0.03 MB DOC) [file pbio.1000543.s012.doc]

| *Mating type* | *Total*  *Mice* |  | *Resulting genotypes* [gene copy number within this genomic interval] | | |  |  |
| --- | --- | --- | --- | --- | --- | --- | --- |
|  |  | *Df(11)17/+*  [1n] | | *Dp(11)17/+*  [3n] | Wild type  [2n] | | *Df(11)17/Dp(11)17*  [2n] |
| ***Dp(11)17/+ X wt*** |  |  | |  |  | |  |
| # mice born | 156 | - | | 86 | 70 | | - |
| % of mice born/ % expected |  | - | | 55/50 | 45/50 | | - |
| ***Df(11)17/+ X wt*** |  |  | | | | | |
| # mice born | 214 | 65 | | - | 149 | | - |
| % of mice born/ % expected |  | 30/50* | | - | 70/50* | | - |
| ***Df(11)17/+ X Dp(11)17/+*** |  |  | | | | | |
| # mice born | 186 | 36 | | 60 | 49 | | 41 |
| % of mice born/ % expected |  | 19.5/25 | | 32.5/25 | 26/25 | | 22/25 |
