# Supplementary material for: Phenotypic Consequences of Copy Number Variation: Insights from Smith-Magenis and Potocki-Lupski Syndrome Mouse Models
Source: PLoS Biol. 2010 Nov 23;8(11):e1000543. doi: 10.1371/journal.pbio.1000543 (PMC2990707; doi:10.1371/journal.pbio.1000543)
Supplement: Table S3 — Genes and quantitative PCR assays before and after recombination in three tissues. (0.06 MB DOC) [file pbio.1000543.s014.doc]

| **Gene** | **Cerebellum^1^** | **Kidney^1^** | **Testis^1^** | **Position** | **Forward primer sequence** | **Reverse primer sequence** |
| --- | --- | --- | --- | --- | --- | --- |
| *Akap1* | + | + | + | chr11:88692106-88712817 | GCCATGAGTGAGATGACAGGCA | GCTTGTCACCTGGGCCAAC |
| AK018772 | + | + |  | chr11:84339186-84349332 | GGCGACTAGCAGCGAGATGT | AAGGCCATCTCCAAACCTGG |
| *Aldoc* | + | + | + | chr11:78137700-78140262 | TGTTACAGAGAAGGTCCTGGCTGC | CATGATGGTCACTCAGGGCCT |
| *Ccl5* |  | + | + | chr11:83339281-83344020 | TTGCAGTCGTGTTTGTCACTCG | TCTGGGTTGGCACACACTTG |
| *Cuedc1* | + | + | + | chr11:87912811-88006420 | CTGCAGCGAAACCGGGAC | TTTCAATCGGTCTCTCTCCAGG |
| *Gdpd1* | + | + | + | chr11:86847296-86887639 | TGATTGGCTGGTGACAACACTG | CATGTGGCTCATGCTAAACACGC |
| *Lhx1* | + | + | + | chr11:84332881-84339036 | CTCGGCCACCACAGGCA | GGATCTTGGGAATCCGGAGATA |
| *Med13* | + |  |  | chr11:86079217-86171027 | TGGCCTCAGTCTCCAGCAATG | CCGCCCAACAGTTACTTGAGACAT |
| *Mrm1* | + | + | + | chr11:84626563-84633017 | GTGGCCACAGGTATTCTTCTACACTC | GGAAACCCTTCTTCTGACTGCA |
| *Phf12* | + | + | + | chr11:77796318-77844035 | AGAGTGTCATCAGGCGCCGC | CACTTGGCTCTTCATCCTGTTTCTGG |
| *Rffl* | + | + | + | chr11:82617322-82642545 | GCCTTATCTCCCTACCTCCTGTACA | TGCTAACCACAGCCATCAGTGT |
| *Rnf43* | + | + |  | chr11:87476589-87549041 | GAAGGCAGAGGA GGCACAACT | GCTCCAAGAACCTCTCAGAGAGGA |
| *Sept4* | + | + | + | chr11:87394633-87404041 | CAACAACACAGAGTGCTGGAAGC | AACTGCTGGTCGATGTATTCGG |
| *Slc13a2* |  | + | + | chr11:78210778-78235687 | TGTCTGATATGGCCCGTGCAG | GTCAGCACTCCGATGATATTGAGC |
| *Spag5* | + | + | + | chr11:78115093-78135955 | AGTATCTGAGCCATAGGCACATCCTG | TGTCAGAGCGCCGAAGATTCT |
| *Taf15* | + | + | + | chr11:83286610-83320242 | TGCAATGAGCCTAGACCAGAGG | CCGGAAATCTCCCCCTGAG |
| *Tmem98* | + | + | + | chr11:80623917-80635535 | AGCTCCTGGATGCACGGACAA | CCAAGTGACTAACGGACAGCAGC |
| *Ypel2* | + |  |  | chr11:86749927-86807264 | AAATCTGTGAAGAGAGCCAGCG | GCTGCTCCCGTCTCAGAAGC |

^1^A “+” sign indicates that the gene is expressed and was tested in this tissue
