# Supplementary material for: Phenotypic Consequences of Copy Number Variation: Insights from Smith-Magenis and Potocki-Lupski Syndrome Mouse Models
Source: PLoS Biol. 2010 Nov 23;8(11):e1000543. doi: 10.1371/journal.pbio.1000543 (PMC2990707; doi:10.1371/journal.pbio.1000543)
Supplement: Table S6 — Genes with abnormal expression in the compound heterozygous mice that can putatively explain the phenotypes found in Df(11)17/Dp(11)17 . (0.05 MB DOC) [file pbio.1000543.s017.doc]

| **Gene name** | ***Gene Symbol*** | **Associated phenotype and cellular function** | **Chromosomal position** |
| --- | --- | --- | --- |
| Calcitonin/calcitonin-related polypeptide, α | *Calca* | Possibly involved in major depression disorder. Hypothesized role in noradrenergic neurotransmission in the CNS | chr7 F1|7 54.0 cM |
| Dynactin 4 | *Dctn4* | Neurodegenerative disease in humans and mice. Related to microtubule motility. | chr18 D2 |
| Glutamate receptor, ionotropic, AMPA1 (α1) /// similar to Glutamate receptor, ionotropic, AMPA1 (α1) | *Gria1* | Associated with bipolar disorders and schizophrenia in humans and related to impaired spacial memory in mice. | chr11 B1.3|11 31.0 cM |
| Histone deacetylase 10 | *Hdac10* | Histone deacetylase inhibitors increase neuronal differentiation in adult forebrain precursor cells. | chr15 E3 |
| Musashi homolog 2 (Drosophila) | *Msi2* | Rna-binding protein Musashi2 has developmentally regulated expression in neural precursor cells and subpopulations of neurons in mammalian CNS. | chr11 B5-C |
| Neuregulin 3 | *Nrg3* | Variants have been linked to susceptibility to schizophrenia and bipolar disorder. | chr14 B |
| γ-aminobutyric acid (GABA-A) receptor, pi | *Gabrp* | Strong evidence for GABA system involvement in schizophrenia. | chr11 A4 |
| Protocadherin 19 | *Pcdh19* | Calcium binding protein. X-linked protocadherin 19 mutations cause female-limited epilepsy and cognitive impairment. | chrX E3 |
| Huntington disease gene homolog | *Hdh* | Huntington disease in humans. | chr5 B2|5 20.0 cM |
| Solute carrier family 18 (vesicular monoamine), member 2 | *Slc18a2 (Vmat2)* | VMAT2 has neuroprotective effects against *N*-methyl-4-phenyltetrahydropyridine (MPTP) toxicity in vivo and in vitro. MPTP induces a persistent Parkinsonian syndrome. Transporter that moves cytoplasmic dopamine into synaptic vesicles for storage and subsequent exocytotic release. | chr19 D3 |
| Ceruloplasmin | *Cp* | Associated with obsessive compulsive disorder and elevation may indicate Alzheimer’s disease. | chr3 D |
| Wolf-Hirschhorn syndrome candidate 1 (human) | *Whsc1* | Protein candidate in Wolf-Hirschhorn syndrome which is characterized by mental retardation, development retardation and muscular hypotonia. | chr5 B1 |
| Regulator of G-protein signaling 4 | *RGS4* | RGS4 polymorphisms predict clinical manifestations in patients with schizophrenia. | chr1 H3|1 86.5 cM |
| Dopa decarboxylase | *Ddc* | Cause aromatic l-amino acid decarboxylase deficiency. Enzyme implicated in the synthesis of dopamine and serotonin. | chr11 A1-A4|11 7.0 cM |
| Integrin alpha V | *Itgav* | Integrin alpha v mediates the effects of GDNF on motor activity in rats. | chr2 D|2 46.0 cM |
| Melanin-concentrating hormone receptor 1 | *Mchr1* | Mchr1 antagonists enhance social recognition. MCH plays an important role in the regulation of stress and emotion. | chr15 E1 |

**Table S6**. Genes with abnormal expression in the compound heterozygous mice which can putatively explain the phenotypes found in *Df(11)17/Dp(11)17*.
