# Supplementary material for: Phenotypic Consequences of Copy Number Variation: Insights from Smith-Magenis and Potocki-Lupski Syndrome Mouse Models
Source: PLoS Biol. 2010 Nov 23;8(11):e1000543. doi: 10.1371/journal.pbio.1000543 (PMC2990707; doi:10.1371/journal.pbio.1000543)
Supplement: Text S1 — Online supplementary text and online supplementary materials and methods. (0.20 MB DOC) [file pbio.1000543.s018.doc]

## Text S1

**Phenotypic consequences of copy number variation: insights from Smith-Magenis and Potocki-Lupski syndrome mouse models**

Ricard *et al.*

**Supplementary text**

*Embryonic survival*

Mice in a C57BL/6-*Tyrc-Brd* pure genetic background were analyzed in order to control for nonlinked modifier loci and minimize the impact of genetic background on phenotype. When animals harboring the duplication were mated to wildtype animals (*Dp(11)17/+* X wt; i.e. wild type or +/+), both genotypes were obtained at the expected Mendelian ratios [86/156 were *Dp(11)17/+* mice, (55%, *P*= 0.2)]. However, from matings of deficiency animals (*Df(11)17/+* X wt) we observed reduced viability of *Df(11)17* mice [65/214, were *Df(11)17/+* animals (30%, *P*< 9.3 E-09)]. When *Dp(11)17/+* X *Df(11)17/+* heterozygous mice were mated, the genetically balanced *Df(11)17/Dp(11)17* mice were obtained at the anticipated proportion [41/186 were compound heterozygous (22%,*P*=0.07)], suggesting that the restoration of gene copy number in this genomic interval is sufficient to normalize embryonic survival (**Supplementary Table S1**).

*Craniofacial abnormalities*

One of the characteristic physical features of SMS patients, the presence of craniofacial abnormalities [1,2] was recapitulated in the SMS mouse model [3,4]. Within this study craniofacial abnormalities were found in 99% of *Df(11)17/+* mice (N= 101), however 0% were observed in +/+ (N=268), *Dp(11)17/+* (N=146) or *Df(11)17/Dp(11)17* (N=41) mice. Skeletal analyses showed that the abnormally upward nasal bone observed in the *Df(11)17/+* mice was not present in any of the other genotypes. Relative distances were measured utilizing different skull landmarks [5]. For all sets of measurements the ANOVA test gave a significant difference between groups. *Post hoc* analysis showed that in all cases the *Df(11)17/+* mice were different from the +/+ mice. *Df(11)17/+* mice had a significantly shorter overall skull and a broader and shorter snout and nasal bone when compared to the +/+ (*P*<0.05 for all measurements) (**Supplementary** **Figure S2**). A lateral view shows that the skull is broader for the *Df(11)17/+* heterozygous mice compared with the +/+. None of these differences were found in *Df(11)17/Dp(11)17* animals, demonstrating that the craniofacial phenotype in these mice is dependent on gene dosage.

*Seizures*

About 20% of SMS patients manifest overt seizures [2,6]. Consistent, with the notion that *RAI1* (**GeneID:** 10743) is the major dosage-sensitive gene within this genomic interval [7-10], one third of homozygous *Rai-/-* mice show seizures by three month of age whereas only 2% of heterozygous *Rai1+/-* mice have overt seizures at 4 month of age [11]. Overt seizures were reported in 20% of *Df(11)17/+* mice in a mixed genetic background [3]. In this study we observed overt seizures in 9% of *Df(11)17/+* mice (9/101), whereas no seizures were seen in *Df(11)17/Dp(11)17* mice (0/41) (**Table 1**).

*Body weight and abdominal fat*

*Df(11)17/+* and *Dp(11)17/+* mice have marked body weight differences. We previously reported that compound heterozygous *Df(11)17/Dp(11)17* mice in a mixed genetic background had normal body weight [3]. In this study we again found that the total body weight for males of the different genotypes at 3 month of age was significantly different between groups (*F*_3, 30_=11.67, *P*<0.0001). *Post hoc* analysis revealed that there were significant differences between *Df(11)17/+* and *Dp(11)17/+* mice when compared to +/+ littermates (27 +/- 0.95g (*P*= 0.005), 21.8 +/-0.5g (*P*= 0.007), and 24.2 +/-0.4 respectively). The total amount of abdominal fat was also significantly different between groups (*F*_3, 30_=12.7, *P*<0.0001) being 0.7 +/- 0.1 g for *Df(11)17/+* (*P*= 0.006), 0.23 +/- 0.015 g for *Dp(11)17/+* (*P*<0.0001), and 0.47 +/- 0.04 g for +/+ animals comprising ~3%, 1% and 2% of the total body weight, respectively. Importantly, we found that *Df(11)17/Dp(11)17* and +/+ animals showed a similar body weight (24.2 +/-0.4g, *P*>0.05) and abdominal fat content (0.46 +/- 0.04 g, *P*>0.05) indicating that this phenotype is corrected with restoration of the normal gene dosage within the syntenic SMS/PTLS genomic interval (**Supplementary Figure S3**).

*Neuromotor activity*

While performing the plus maze test, we noticed that 5 out of 23 *Df(11)17/+* mice either hang or fall from the maze, while only three of the other tested mice (n=71) of any other genotype did so. This prompted us to study neuromotor skills in the four different genotypes. During the first day the Dowel test for ataxia was conducted. Significant overall difference was found in the total number of falls (*F*_(3, 41_= 8.22; *P*< 0.001). *Post hoc* analysis revealed significant differences between the *Df(11)17/+* and +/+ mice (*P*=0.01). No differences were found between *Df(11)17/Dp(11)17* and +/+ mice (*P*>0.05) (**Supplementary Figure S1A**). Next we measured the amount of time mice can suspend themselves from a wire before falling. Significant differences were found overall (*F*_(3,48)=_3.8, *P*= 0.16). *Post hoc* analysis showed that *Df(11)17/+* mice can suspend themselves for shorter periods of time when compared to the +/+ littermates (*P*=0.04; **Supplementary Figure S1B**). There is no significant difference between +/+ and *Df(11)17/Dp(11)17* mice (*P*>0.05). Finally, the rotarod test was performed. Mice were placed on a rotating rod and the speed of rotation was gradually increased (in 4 trials per day during 4 consecutive days). When *Dp(11)17/+* mice were compared with +/+ mice (**Supplementary Figure S1C**) we found that both genotypes could stay on the rotating rod for a similar amount of time, and performed similarly during the first three days. On the fourth day *Dp(11)17/+* mice tended to fall from the rod significantly sooner than wild type. *Df(11)17/+* mice showed a significantly impaired performance when compared to the *+/+* littermates during the first day, however, they learned how to walk on the rotating rod, within the second day (**Supplementary Figure S1D**). No differences were observed when *Df(11)17/Dp(11)17* mice were compared to the *+/+* littermates (**Supplementary Figure S1E**). Overall these results indicate that the altered neuromotor function in *Df(11)17/+* mice is due to gene dosage within this genomic interval (**Table 1**).

*Social interaction assessment using the tube test*

To evaluate social interactions, we used the tube test [12], in which two mice of different genotypes are released at the opposite end of an acrylic tube and the one who backs out is recorded. The mouse remaining in the tube is declared the winner. In this study *Dp(11)17/+* mice, when confronted to +/+ mice, backed out 10% of the times (1/10) in the first round, and 0% of the times (0/10) in the second round, in agreement with previous results [12]. When *Df(11)17/+* mice were tested against +/+ mice they backed out 90% (9/10) of the experimental trials in the first round, and 100% of the trials (10/10) in the second round revealing a diametrically opposing phenotype to that observed for *Dp(11)17/+* mice*.* Interestingly when *Df(11)17/Dp(11)17* mice were confronted to *+/+* mice they backed out of the tube 50% of the times in round one (5/10) and 40% in round two (4/10), clearly indicating that this is a phenotype directly related to gene copy number within this interval (**Supplementary Figure S4 and Table 1**).

These different experiments indicate that embryonic survival, craniofacial abnormalities, overt seizures, neuromotor activity, social interaction (as measured by the tube test) and both body weight and fat composition result from CNV gene dosage effects because balancing the genotypes abolishes the abnormal trait (**Table 1**).

*Rai1 +/- and Df(11)17/+*

*Rai1*+/- mice show phenotypes similar to that of *Df(11)17/+* mice, especially in obesity and craniofacial abnormalities, clearly indicating that the dosage change of *RAI1/Rai1* is a major factor in the SMS phenotypes. The neurobehavioral features of *Rai1*+/- mice have not been described in detail on a pure background (like in this study). However on a mixed N2 background, *Rai1*+/- mice showed much less behavioral abnormalities than their counterparts with the engineered deletion. On both mixed and pure (C57BL/6) background, a lower penetrance of the craniofacial features compared to *Df(11)17* mice was reported, suggesting the existence of other genes/modifiers in the SMS/PTLS interval that also contribute to the phenotypes [4,11,13,14].

*Altered gene expression in Df(11)17/Dp(11)17 mice.*

As some neurobehavioral manifestations appear to be influenced by genomic structural changes without changes in gene dosage, we sought to identify gene(s) that are modified in their relative expression levels in the *Df(11)17/Dp(11)17* mouse. Thus, we compared the transcriptome of the hippocampus and the cerebellum of these mice with that of +/+ littermates by microarray analyses. For both tissues we ranked the most differentially expressed transcripts between both genotypes (**Supplementary Table S4 and S5**). All but four of the transcripts mapping to the engineered interval (*AI662501*, *AW215868*, *BE947384* and *Smcr8* (**GeneID:** 237782)) present similar relative expression levels between genotypes (i.e. not present in the first 250-ranked differentially expressed genes) demonstrating that gene copy number restoration is paralleled by normalization of expression levels.

Gene ontology analysis of the data indicates that candidate pathways to be involved in features of *Df(11)17/Dp(11)17* mice include detection of stimulus (GO0051606). We noticed, for example, an underexpression of the genes encoding guanine nucleotide binding proteins, such as the *alpha transducing 2* (*Gnat2;* GeneID: 14686) and the *tubby-like protein 1* (*Tulp1;* GeneID: 22157) in the hippocampus. These proteins are involved in cell signaling and visual perception as well as neuronal differentiation and development [15]. They are associated with several mammalian diseases such as obesity, retinal degeneration, and hearing loss. On the contrary, we recorded an overexpression of the *neuropeptide FF receptor 2* (*Npffr2;* GeneID: 104443) gene, the product of which plays a role in neuronal regulation under both physiological and pathological conditions. *Npffr2* is upregulated in an inflammatory pain state, while its ligand, Npff, attenuates stress-induced analgesia in rodents [16]. Interestingly, its binding can be inhibited by guanine nucleotides, suggesting that NPFF elicits its actions through G protein-coupled receptors.

In the cerebellum, we observed the overexpression of the gene encoding the *calcitonin/calcitonin-related polypeptide alpha* (*Calca;* GeneID: 12310), possibly involved in major depression disorder [17]. It has been hypothesized that *Calca* plays a role in noradrenergic neurotransmission in the CNS [18]. Likewise, we found that the *tachykinin 1* gene (*Tac1*; GeneID: 21333), encoding a neuropeptide G protein that indirectly modulates GABA release and synaptic transmission of sensory perception of pain [19], was overexpressed in the compound heterozygote. The enhanced release of endogenous glutamate and aspartate by tachykinins and calcitonin gene-related peptide has important physiological implications to strengthen synaptic connections. Other genes that show perturbed expression encode proteins related to calcium binding, signal transduction, homeostasis and ion transport, regulation of transcription and G-proteins and G-proteins-coupled receptors. More specifically, we found altered expression for *Ndel1* (GeneID: 83431), *Mpp4* (227157), *Neurod4* (11923), *neuregulin 3* (*Nrg3*; 18183)[20] and *reticulon 4 receptor-like* (*Rtn4rl2*; 269295), all genes related to nervous system development and differentiation. **Supplementary Table S6** summarizes the genes that show perturbed expression in the compound heterozygote and were previously related to neurological conditions.

**Supplementary Materials and Methods**

*Ethics Statement*

All behavioral testing procedures were approved by the CECS Institutional Animal Care and followed the National Institutes of Health Guidelines, ‘Using Animals in Intramural Research’.

*Mice strain*

The generation of the heterozygous *Dp(11)17/+* and *Df(11)17)/+* mice carrying the duplication and its reciprocal deletion, respectively, using AB2.2 ES cell derived from a 129S5/SvEvBrd mouse (129S5) is described in [3]. The engineered animals were backcrossed with C57BL/6J-*Tyr^c-Brd^* mice for twelve generations. Mice were genotyped visually by the presence of Agouti coloration in the coat color and by PCR. A third strain, *Df(11)17/Dp(11)17*, that carries the deletion on one allele and the duplication of the other, was then generated by crossing *Dp(11)17/+* and *Df(11)17)/+* animals. A schematic view of the different genotypes is shown in Figure 1. The engineered ~1.6 Mb rearrangements include at least 34 loci, which we name “SMS/PTLS genes”, i.e. *Cops3* (GeneID: 26572)*,* *Nt5m* (103850)*, Med9* (192191)*, Rasd1* (19416)*, Pemt* (18618), *Rai1* (19377)*, Srebf1* (20787)*, Tom1l2* (216810), *Lrrc48* (74665)*, Atpaf2* (246782)*, 4933439F18Rik* (Ensembl:ENSMUSG00000018415)*, Drg2* (GeneID: 13495)*, Myo15* (17910)*, Alkbh5* (268420)*, AW215868, Llgl1* (16897)*, Flii* (14248)*, Smcr7* (237781)*, Top3a* (21975)*, Smcr8* (237782)*, Shmt1 (*20425)*, Dhrs7b* (216820)*, Tmem11* (216821)*, Gtlf3b* (24083)*, Gtlf3a, Map2k3* (26397)*, Kcnj12* (16515)*, Tnfrsf13b* (57916)*, Usp22* (216825), *Aldh3a1* (11670)*, Aldh3a2* (11671)*, Slc47a2* (380701)*,* *Slc47a1* (67473) and *Zfp179* (22671; a.k.a. *Rnf112*). Their expression levels are monitored by a set of 70 probesets (transcripts; see below).

As maintenance of the original genetic background on the engineered chromosome has been reported even after 10 backcrossing generations [21] we genotyped the entire length of mouse chromosome 11 (MMU11) in *Dp(11)17/+* and *Df(11)17)/+* mice. Potential SNPs were identified by comparing the genotype of the C57BL6/J and 129S1/SvImJ strains over the 8.27 million SNPs published in [22]. These were then genotyped in the SMS and PTLS mice models, 129S2 (129S2/SvPas), 129S5 and C57BL6/J animals by direct sequencing. These analyses showed that the region proximal from the engineered interval had recombined up to coordinate 55,081,047 (4.5 Mb centromerically from the engineered cassette) in both models of genomic disorders. On the other hand, the distal section had either only partially or not recombine at all to the C57BL6/J background in *Dp(11)17/+* and *Df(11)17)/+*, respectively. The retention of large blocks of genetic material from the 129S5 parental strain in these mice has the potential to affect the gene expression profile of genes from the same chromosome. For example, sequence variants can have significant impact on hybridization to expression arrays that are designed on the basis of the C57BL/6J reference genome sequence [23-25]. We thus devised a strategy to take this fact into account and discard the transcripts that could possibly be influenced by their 129S5 background.

Genotyping of the entire length of MMU11 in 129S2 and 129S5 mice showed also that these strains were genetically identical from the SMS/PTLS engineered interval to the telomere. Thus we used expression data previously established in our laboratory with the same microarray platform (Affymetrix GeneChip Mouse Genome 430 2.0 arrays) and that compare 129S2 and C57BL6/J male animals (GEO Series accession number: GSE10744)[26]) to identify the transcripts that show different level of expression and thus that should be removed from our analysis (see gene expression profiling and data analysis sections below for details).

Genotyping of *Dp(11)17/+* animals showed that the 16 megabases MMU11:76843886-92963733 interval recombined to the C57BL/6J genetic background between the 12^th^ and 17^th^ backcross (**Supplementary Figure S2A**).

*Gene expression profiling*

Whole hippocampus, cerebellum, testis, kidney, heart, cortex, liver, lung from *+/+*, *Dp(11)17/+*, *Df(11)17/+* and *Df(11)17/Dp(11)17* adults were dissected and immediately frozen on dry ice. Total RNA was extracted using TRIzol reagent (Invitrogen, Carlsbad, NM, USA) and cleaned on RNeasy columns (Qiagen). RNA quantity and quality were assessed by NanoDrop®ND-1000 spectrophotometer and Agilent 2100 bio-analyzer chips, respectively. The synthesis of cRNA was performed using the GeneChip® One-Cycle cDNA Synthesis (for cerebellum, testis, kidney and heart) and the MessageAmp™ II-Biotin Enhanced Single Round aRNA Amplification (hippocampus) kits according to the manufacturers' protocols (Affymetrix, Santa Clara, CA, USA and Applied Biosystems/Ambion, Austin, TX, respectively). Labeling, hybridization and scanning of the samples were performed as described by Affymetrix (www.affymetrix.com). GeneChip Mouse Genome 430 2.0 arrays (Affymetrix; Santa Clara, CA, USA), each interrogating 45,101 target sequences with appropriate probesets, were used to hybridize the labelled cRNA of hippocampus, cerebellum, testis, kidney and heart. At least three male individuals of each of the four genotypes were processed. These data have been deposited in NCBIs Gene Expression Omnibus (GEO, http://www.ncbi.nlm.nih.gov/geo/) and are accessible through GEO Series accession number GSE14802. Expression data analysis was performed in R using the bioconductor package for low-level analysis and normalization ([http://www.R-project.org](http://www.pubmedcentral.nih.gov/redirect3.cgi?&&auth=0ckzSSSfwhdQtejeA476-haH8Qjl_NP7BPc5osQ8H&reftype=extlink&artid=1160250&iid=119861&jid=4&FROM=Article|Body&TO=External|Link|URI&article-id=1160250&journal-id=4&rendering-type=normal&&http://www.R-project.org) [27]). Hybridization quality was assessed using Bioconductor “affy” and “affyPLM” packages [27,28]. Normalized expression signals were calculated from Affymetrix CEL files using RMA [28,29]. Differential hybridized features were identified using the Bioconductor package “limma” that implements linear models for microarray data [30]. In “limma”, *P* values are obtained from moderated t-statistics or F-statistics using Empirical Bayesian methods. *P* values are then adjusted for multiple testing with Benjamini and Hochberg’s method to control the false discovery rate (FDR) [31]. For each tissue, these expression data allowed clustering of the samples by genotype (not shown).

*Expression Data Analysis*

We compared the results obtained within two different sets of data. The first set of data, which we named Most-diff, is composed of the 41,563 target sequences that matched unambiguously to the mouse genome (February 2006 assembly– Mm8) out of a total of 45,101 interrogated. To obtain the second set, which we named Most-diff-restricted, we subtracted from the first set the 15 and 114 transcripts that map to the engineered interval or the distal portion of MMU11, respectively, are differentially expressed amongst *+/+*, *Dp(11)17/+*, *Df(11)17/+* and *Df(11)17/Dp(11)17* animals in at least one of the five assessed tissue and are differentially expressed between 129S2 and C57BL6/J (False discovery rate < 0.1; corrected for multiple testing) in at least one of six tissues (brain (33 transcripts), liver (44), testis (58), kidney (51), lung (46), and heart (19)) using transcriptome profilings previously established in our laboratory (GEO Series accession number: GSE10744)[26]. We note that three of the tissues assessed are the same as the one profiled for the present study and that brain comprises a fourth one (i.e. hippocampus). The cumulative contribution of each tissue to the list of differentially expressed MMU11 transcripts is presented in **Supplementary Figure S7**. It suggests that the vast majority of the transcripts that are differentially expressed between the two above strains are identified with this set of six major tissues. The strategy we devised to identify genes to be discarded as potentially influenced by the genetic background is conservative, as we are assessing expression in a homozygous background while *Dp(11)17/+* and *Df(11)17/+* mice are heterozygote.

For each dataset, we ranked the expressed target sequences according to the *P* value of the F-test performed on the four genotypes and retained for each tissue the 10,000 most differentially expressed transcripts. For each expressed transcripts we calculated the fold change between the three engineered genotypes (*Dp(11)17/+*, *Df(11)17/+* and *Df(11)17/Dp(11)17*) and the wild type strain (*+/+*). To construct the heatmaps, we extracted amongst 1122 (Most-diff set) and 1056 transcripts (Most-diff-restricted set) with an F-test adjusted *P* value ≤ 0.1, the ones that map to mouse chromosome 11. We performed the bioinformatic analyses using Perl (<http://www.perl.org/>) and R scripts (http://www.r-project.org/). The data are stored in a MySQL database (<http://www.mysql.com/>).

*Real-Time Quantitative PCR*

Taqman real-time quantitative polymerase chain reaction was performed as published [12,32]. Briefly, male whole hippocampus and cerebellum and female whole hippocampus, cerebellum, cortex, liver and lung total RNA was converted to cDNA using Superscript III (Invitrogen) primed with a mix of oligo(dT) and random hexamers. Oligos and probes were designed using the PrimerExpress program (Applied Biosystem) with default parameters (**Supplementary Table S2**). Non intron-spanning assays were tested in standard +/– RT reactions of RNA samples for genomic contamination. HPLC-purified Yakima-Yellow Dark-QUENCHER-labeled double-dye Taqman probes and qPCR mastermix (RT-QP2X-03) were obtained from Eurogentec (Seraing). The efficiency of each Taqman assay was tested in a cDNA dilution serie as described [33]. All RT-PCR reactions were performed in a 10-μl final volume and three replicates per sample and set up in a 384 wells plate format using a Freedom EVO robot (TECAN) and run in an ABI 7900 Sequence Detection System (Applied Biosystems) with the following amplification conditions: 50°C for 2 min, 95°C for 10 min, and 50 cycles of 95°C 15 sec/60°C for 1 min. Each plate included the appropriate normalization genes to control for any variability between the different plate runs. Raw threshold cycles (Ct) values were obtained using SDS2.2 (Applied Biosystems). To calculate the normalized relative expression ratio between aneuploid mice and normal littermates, we followed the method described in [12,32], and exploited the geNorm method to select normalization genes [34].

To directly assess the level of expression changes due to linkage disequilibrium between the engineered interval and flanking 129S5 polymorphisms, we compared relative level of expression in different backcrosses, which differ by the extent of the recombined region using quantitative PCR. Specifically, the MMU11:76843886-92963733 interval is recombined in *Dp(11)17/+* animals from the 17^th^, but not in those from the 12^th^ backcross. The list of assessed genes and the assays used are presented in **Supplementary Table S3**. They are mapping to the above-defined region and show significant differences in expression between *Dp(11)17/+* and *+/+* in the microarray profiling experiments.

*Clustering, CpG islands, ubiquity and level of expression*

To assess the clustering of the affected transcripts along mouse chromosomes we used a modified version of the method described in [35]. With this method we test statistically whether the observed regional differences in the density of most differentially expressed transcripts in the Most-diff-restricted set represent real differences (SMS-PTLS region excluded). Significance levels are determined by randomly sampling the same number of transcripts in the same length of the sequence and calculating the statistic for each of 10,000 permutations to build the null distribution as described in [36]. Instead of determining the largest differential of density of variable sites, we determined the sum of differentials for the entire length of the sequence and estimated *P*-values using the same metric for the permutations as implemented in [37]. This modification makes the test more sensitive to numerous but small clusters of affected transcripts.

CpG islands locations were retrieved from the UCSC genome browser (Mm8 assembly). To assess if the MMU11 transcripts that show modified expression levels in the four mouse genotypes mapped in proximity of CpG islands, we determined the proportion of the 136 MMU11 transcripts from Most-diff-restricted (SMS-PTLS region excluded) most differentially expressed (i.e. those with a F-test adjusted *P* value < 0.2) that overlapped such an island. We found that 53 out of 136 transcripts overlap a CpG island (39 %) in Most-diff-restricted (98 out of 250 in Most-diff). To evaluate if this fraction was statistically significant, we randomly picked 10,000 times 136 of the 3051 expressed transcripts mapping to MMU11 outside of the SMS-PTLS region and assessed if they were overlapping a CpG island.

We gauged if affected transcripts were ubiquitously expressed by comparing the number of tissues in which the 136 MMU11 most differentially expressed and the 3051 MMU11 expressed transcripts (SMS-PTLS region excluded) were identified.

To evaluate if highly expressed transcripts are more likely to be differentially expressed in the different mice genotypes and thus could bias our results, we assessed for each genotype if there was a relation between the level of expression measured on the array and the F statistic value. We found that the most differentially expressed transcripts were not more prone to be expressed at higher or lower levels than other transcripts (**Supplementary Figure S4**).

*Mouse phenotyping*

At weaning age, male mice were grouped by genotypes and housed two to four per cage in a room with a 12 h light:dark cycle (lights on at 7 AM, off at 7 PM) with access to food (Teklad Global 19% Protein Extruded Rodent Diet from Harlan) and water *ad lib*. Behavioral testing was performed between 9 AM and 1 PM. All behavioral testing procedures were approved by the CECS Institutional Animal Care and followed the National Institutes of Health Guidelines, ‘Using Animals in Intramural Research’. At 10 weeks of age, mice were tested with a battery of test to assay for anxiety and social behaviors or motor skills. For each test, the number of mice (n) tested is indicated in the respective figure caption. At exactly 12 weeks of age some mice were sacrificed, weighed and utilized for skeletal preparation as described previously [3].

*Phenotyping anxiety and social behaviors.*

With 0–2 days between each test we performed (i) the elevated plus maze, (ii) the sociability and preference for social novelty, and (iii) the dominance tube test. The elevated plus maze (50 cm above the floor) consists of two closed arms (with 20 cm walls high) and two open arms (without walls). Each arm is 33 cm long. The light level at the center of the maze was ~300 lux. Animals were placed in the center of the maze and allowed to freely explore in a 5 min trial. The position of the animal was recorded every 10 s having a total of 30 observations/mouse. The percentage of time for the mice in the open (or close) arm was estimated as: number of observations in the open (or close) arm x 100/total observations. Mice that felled from the maze were excluded from the statistical analyses.

The social behavior apparatus consisted of a rectangular three-chambered cage made of clear carbonate [38]. Dividing walls have retractable doors allowing access to each section of the cage. The test consisted of three intervals of 10 min each. In the first 10 min (habituation period), the mouse was placed in the center chamber and allowed to explore the entire cage (doors open) and its’ position was recorded by an observer every 10 s. After the habituation period was finished, the test mouse was enclosed in the center compartment, and an unfamiliar mouse (stranger 1) was placed into a plastic container with openings that allow for visual and olfactory recognition, but prevent direct contact, in one side of the chambers, and an empty container in the other chamber. The doors were open and the position of the test mouse was recorded for another 10 min. To measure the preference for social novelty at the end of the 10 min interval, the mouse was enclosed again in the center chamber and a second unfamiliar mouse (stranger 2) was placed in the empty container. The doors were open again and the position of the tester mouse was recorded for an extra 10 min. Data were analyzed as a percentage of total time spent in each of the chamber sections in each of the 10 min intervals. The stranger mice were adult C57BL/6-*Tyr^c-Brd^* housed far away from the tester mice and habituated to the container for a period of 10 min during 5 days before the test. To exclude any environmental interference within the social test chamber, we evaluated the percentage of observations for each genotype in each compartment during the habituation period, and no chamber preference was evident for any genotype (*P*>0.05). To exclude any environmental interference the stranger 1 and the empty cage were placed alternatively in the left or right side of the test chamber and there was no significant difference in the number of observations with the stranger 1 for both genotypes if it was placed in the right or left compartment of the test chamber (*P* >0.05).

The tube test consisted of a 30 cm long x 3.5 cm diameter tube in which two age-matched males of different genotype were released toward each other from the opposite ends of the tube [12]. A subject was declared a ‘winner’ when its opponent backed out of the tube. All matches resolved within the first 5 min. Each pairing was performed twice, each of the mice entering the tube using alternative ends and one trial followed by the other with no intertrial interval. Mice were trained to enter the tube alternatively at either end prior to the test.

*Phenotyping neuromotor skills*

During the first day the dowel test was performed. The apparatus consists in two elevated platforms (50 cm from the floor) connected by a wooden dowel. The test was divided into three parts. First, the mice were placed on each platform for 1min in order to teach them the safety of the platform. After that, mice were taken and placed on the wooden dowel 10 cm away from one of the platforms and the numbers of falls to reach the platform within the first min were recorded. The mice that reached the platform in less than 1 min were placed on the center of the elevated dowel at 30 cm from platform and the latency time to reach the platform, the number of arrivals and the numbers of falls were recorded during 90 s. Next the hanging ability was tested. The mice were suspended by the tail and placed hanging from a wire. The hanging time was recorded until they fell.

During the following four days the rotarod test was performed. The animals were placed on rotating rod (Ugo Basile) with an accelerating velocity that ranged from 5 to 40 r.p.m. and the time the mouse spends in the rotating rod before falling down was recorded. Each day four consecutive trials were performed of a maximum time of 600 s. The inter-trial time was 20 min.

*Phenotyping statistical analysis*

The craniofacial, weight, plus maze, dowel, hanging ability and the rotating rod data were analyzed using the independent one-way (genotype) ANOVA followed by a Fisher–LSD analysis when a significant *F*-value was determined. Sociability and social novelty preference data were analyzed using two-way (genotype X side) ANOVA with repeated measure (side) followed by a Fisher–LSD analysis when a significant *F*-value was determined.

**Bibliography**

1. Allanson JE, Greenberg F, Smith AC (1999) The face of Smith-Magenis syndrome: a subjective and objective study. J Med Genet 36: 394-397.

2. Greenberg F, Lewis RA, Potocki L, Glaze D, Parke J, et al. (1996) Multi-disciplinary clinical study of Smith-Magenis syndrome (deletion 17p11.2). Am J Med Genet 62: 247-254.

3. Walz K, Caratini-Rivera S, Bi W, Fonseca P, Mansouri DL, et al. (2003) Modeling del(17)(p11.2p11.2) and dup(17)(p11.2p11.2) contiguous gene syndromes by chromosome engineering in mice: phenotypic consequences of gene dosage imbalance. Mol Cell Biol 23: 3646-3655.

4. Yan J, Bi W, Lupski JR (2007) Penetrance of craniofacial anomalies in mouse models of Smith-Magenis syndrome is modified by genomic sequence surrounding Rai1: not all null alleles are alike. Am J Hum Genet 80: 518-525.

5. Richtsmeier JT, Baxter LL, Reeves RH (2000) Parallels of craniofacial maldevelopment in Down syndrome and Ts65Dn mice. Dev Dyn 217: 137-145.

6. Goldman AM, Potocki L, Walz K, Lynch JK, Glaze DG, et al. (2006) Epilepsy and chromosomal rearrangements in Smith-Magenis Syndrome [del(17)(p11.2p11.2)]. J Child Neurol 21: 93-98.

7. Bi W, Saifi GM, Girirajan S, Shi X, Szomju B, et al. (2006) RAI1 point mutations, CAG repeat variation, and SNP analysis in non-deletion Smith-Magenis syndrome. Am J Med Genet A 140: 2454-2463.

8. Slager RE, Newton TL, Vlangos CN, Finucane B, Elsea SH (2003) Mutations in RAI1 associated with Smith-Magenis syndrome. Nat Genet 33: 466-468.

9. Walz K, Paylor R, Yan J, Bi W, Lupski JR (2006) Rai1 duplication causes physical and behavioral phenotypes in a mouse model of dup(17)(p11.2p11.2). J Clin Invest 116: 3035-3041.

10. Zhang F, Potocki L, Sampson JB, Liu P, Sanchez-Valle A, et al. (2010) Identification of uncommon recurrent Potocki-Lupski syndrome-associated duplications and the distribution of rearrangement types and mechanisms in PTLS. Am J Hum Genet 86: 462-470.

11. Bi W, Yan J, Shi X, Yuva-Paylor LA, Antalffy BA, et al. (2007) Rai1 deficiency in mice causes learning impairment and motor dysfunction, whereas Rai1 heterozygous mice display minimal behavioral phenotypes. Hum Mol Genet 16: 1802-1813.

12. Molina J, Carmona-Mora P, Chrast J, Krall PM, Canales CP, et al. (2008) Abnormal social behaviors and altered gene expression rates in a mouse model for Potocki-Lupski syndrome. Hum Mol Genet 17: 2486-2495.

13. Bi W, Ohyama T, Nakamura H, Yan J, Visvanathan J, et al. (2005) Inactivation of Rai1 in mice recapitulates phenotypes observed in chromosome engineered mouse models for Smith-Magenis syndrome. Hum Mol Genet 14: 983-995.

14. Burns B, Schmidt K, Williams SR, Kim S, Girirajan S, et al. (2010) Rai1 haploinsufficiency causes reduced Bdnf expression resulting in hyperphagia, obesity, and altered fat distribution in mice and humans with no evidence of metabolic syndrome. Hum Mol Genet.

15. Carroll K, Gomez C, Shapiro L (2004) Tubby proteins: the plot thickens. Nat Rev Mol Cell Biol 5: 55-63.

16. Gelot A, Frances B, Roussin A, Latapie JP, Zajac JM (1998) Anti-opioid efficacy of neuropeptide FF in morphine-tolerant mice. Brain Res 808: 166-173.

17. Buervenich S, Xiang F, Sydow O, Jonsson EG, Sedvall GC, et al. (2001) Identification of four novel polymorphisms in the calcitonin/alpha-CGRP (CALCA) gene and an investigation of their possible associations with Parkinson disease, schizophrenia, and manic depression. Hum Mutat 17: 435-436.

18. Kangrga I, Randic M (1990) Tachykinins and calcitonin gene-related peptide enhance release of endogenous glutamate and aspartate from the rat spinal dorsal horn slice. J Neurosci 10: 2026-2038.

19. Cao YQ, Mantyh PW, Carlson EJ, Gillespie AM, Epstein CJ, et al. (1998) Primary afferent tachykinins are required to experience moderate to intense pain. Nature 392: 390-394.

20. Chen PL, Avramopoulos D, Lasseter VK, McGrath JA, Fallin MD, et al. (2009) Fine mapping on chromosome 10q22-q23 implicates Neuregulin 3 in schizophrenia. Am J Hum Genet 84: 21-34.

21. Gerlai R (1996) Gene-targeting studies of mammalian behavior: is it the mutation or the background genotype? Trends Neurosci 19: 177-181.

22. Frazer KA, Eskin E, Kang HM, Bogue MA, Hinds DA, et al. (2007) A sequence-based variation map of 8.27 million SNPs in inbred mouse strains. Nature 448: 1050-1053.

23. Alberts R, Terpstra P, Li Y, Breitling R, Nap JP, et al. (2007) Sequence polymorphisms cause many false cis eQTLs. PLoS One 2: e622.

24. Benovoy D, Kwan T, Majewski J (2008) Effect of polymorphisms within probe-target sequences on olignonucleotide microarray experiments. Nucleic Acids Res 36: 4417-4423.

25. Walter NA, McWeeney SK, Peters ST, Belknap JK, Hitzemann R, et al. (2007) SNPs matter: impact on detection of differential expression. Nat Methods 4: 679-680.

26. Henrichsen CN, Vinckenbosch N, Zollner S, Chaignat E, Pradervand S, et al. (2009) Segmental copy number variation shapes tissue transcriptomes. Nat Genet 41: 424-429.

27. Gautier L, Cope L, Bolstad BM, Irizarry RA (2004) affy--analysis of Affymetrix GeneChip data at the probe level. Bioinformatics 20: 307-315.

28. Bolstad BM, Irizarry RA, Astrand M, Speed TP (2003) A comparison of normalization methods for high density oligonucleotide array data based on variance and bias. Bioinformatics 19: 185-193.

29. Irizarry RA, Hobbs B, Collin F, Beazer-Barclay YD, Antonellis KJ, et al. (2003) Exploration, normalization, and summaries of high density oligonucleotide array probe level data. Biostatistics 4: 249-264.

30. Smyth GK (2004) Linear models and empirical bayes methods for assessing differential expression in microarray experiments. Stat Appl Genet Mol Biol 3: Article3.

31. Benjamini Y, Hochberg Y (1995) Controlling the false discovery

rate: a practical and powerful approach to multiple testing. Journal of the Royal Statistical Society Series B 57: 289-300.

32. Merla G, Howald C, Henrichsen CN, Lyle R, Wyss C, et al. (2006) Submicroscopic deletion in patients with Williams-Beuren syndrome influences expression levels of the nonhemizygous flanking genes. Am J Hum Genet 79: 332-341.

33. Livak KJ, Schmittgen TD (2001) Analysis of relative gene expression data using real-time quantitative PCR and the 2(-Delta Delta C(T)) Method. Methods 25: 402-408.

34. Vandesompele J, De Preter K, Pattyn F, Poppe B, Van Roy N, et al. (2002) Accurate normalization of real-time quantitative RT-PCR data by geometric averaging of multiple internal control genes. Genome Biol 3: RESEARCH0034.

35. Tang H, Lewontin RC (1999) Locating regions of differential variability in DNA and protein sequences. Genetics 153: 485-495.

36. Reymond A, Marigo V, Yaylaoglu MB, Leoni A, Ucla C, et al. (2002) Human chromosome 21 gene expression atlas in the mouse. Nature 420: 582-586.

37. Dermitzakis ET, Reymond A, Scamuffa N, Ucla C, Kirkness E, et al. (2003) Evolutionary discrimination of mammalian conserved non-genic sequences (CNGs). Science 302: 1033-1035.

38. Nadler JJ, Moy SS, Dold G, Trang D, Simmons N, et al. (2004) Automated apparatus for quantitation of social approach behaviors in mice. Genes Brain Behav 3: 303-314.

**Supplementary Table S1:** The viability of the different genotypes in this inbreed genetic background is dependent on gene dosage. Typical matings between animals *Dp(11)17/+* X +/+, *Df(11)17/+* X +/+ and *Df(11)17/+* X *Dp(11)17/+* mice (12^th^ backcross in C57BL/6-*Tyrc-Brd* genetic background). The total numbers of mice born from each mating type is indicated, plus the resulting n of each genotype. The % of mice born/ % expected for each genotype are showed. The * denotes significantly different from the expected Mendelian ratio. Gene copy number within this genomic interval is indicated in brackets for each genotype.

**Supplementary Table S2: Genes and quantitative PCR assays to validate microarrays experiments**

**Supplementary Table S3: Genes and quantitative PCR assays before and after recombination in three tissues**

**Supplementary Table S4: transcripts differentially expressed in the hippocampus of *Df(11)17/Dp(11)17* mouse.**

**Supplementary Table S5: transcripts differentially expressed in the cerebellum of *Df(11)17/Dp(11)17* mouse.**

**Supplementary Table S6**:

Genes with abnormal expression in the compound heterozygous mice which can putatively explain the phenotypes found in *Df(11)17/Dp(11)17*.
